# Translocating proteins compartment-specifically alter the fate of epithelial-mesenchymal transition in a compartmentalized Boolean network model

**DOI:** 10.1038/s41540-022-00228-7

**Published:** 2022-06-09

**Authors:** Péter Mendik, Márk Kerestély, Sebestyén Kamp, Dávid Deritei, Nina Kunšič, Zsolt Vassy, Péter Csermely, Daniel V. Veres

**Affiliations:** 1 Department of Molecular Biology, Institute of Biochemistry and Molecular Biology, Semmelweis University, Budapest, Hungary; 2 Turbine Ltd, Budapest, Hungary

**Keywords:** Dynamic networks, Cancer, Molecular biology

## Abstract

Regulation of translocating proteins is crucial in defining cellular behaviour. Epithelial-mesenchymal transition (EMT) is important in cellular processes, such as cancer progression. Several orchestrators of EMT, such as key transcription factors, are known to translocate. We show that translocating proteins become enriched in EMT-signalling. To simulate the compartment-specific functions of translocating proteins we created a compartmentalized Boolean network model. This model successfully reproduced known biological traits of EMT and as a novel feature it also captured organelle-specific functions of proteins. Our results predicted that glycogen synthase kinase-3 beta (GSK3B) compartment-specifically alters the fate of EMT, amongst others the activation of nuclear GSK3B halts transforming growth factor beta-1 (TGFB) induced EMT. Moreover, our results recapitulated that the nuclear activation of glioma associated oncogene transcription factors (GLI) is needed to achieve a complete EMT. Compartmentalized network models will be useful to uncover novel control mechanisms of biological processes. Our algorithmic procedures can be automatically rerun on the https://translocaboole.linkgroup.hu website, which provides a framework for similar future studies.

## Introduction

The spatial and temporal organization of intracellular proteins is well regulated and it often defines the cellular fate^1^. Proteins must function specifically at given times with certain functions, thus the eukaryotic cell is organized into different compartments, called organelles^2^. These subcellular organelles must provide a specific chemical environment and a specific set of interaction partners, so that proteins can function physiologically^3^. The physiological distribution of proteins is essential for the life of a cell and it plays an inevitable role in biological processes such as cellular learning^4^.

Protein translocation refers to the regulated change of a protein’s subcellular localization between subcellular compartments^5^. This results in altered interactors and functions of the translocated protein. Disturbances in the regulation of protein translocation lead to altered cellular localization of proteins which may contribute to cellular dysfunction and different pathologies^6,7^. Pathological alterations in the localization of proteins also function as biomarkers for disease progression^8^. Moreover, pharmaceutical targeting of protein translocation is a promising therapeutic strategy^9^.

We have recently assembled the Translocatome database, which is a comprehensive dataset of human translocating proteins^5^. It contains 13,066 human proteins, each characterized with a translocation probability. 34% of the proteins in the Translocatome database were predicted as translocating proteins. This ratio is supported by the experimental results of Thul et al., which shows that approximately 50% of the human proteome is multi-localized^10^. These large datasets provide available data that can be utilized to create in silico models of cellular processes designed in a way that they can represent subcellular dynamics.

Epithelial-mesenchymal transition (EMT) is a biological process which physiologically accompanies early embryonic development but may also be activated pathologically during cancer progression or tissue fibrosis. The reversion of EMT is called mesenchymal-epithelial transition (MET). Thus there is a dynamic transition between the epithelial and mesenchymal phenotypes, and cells may reside in a spectrum of intermediary phases, so-called hybrid states^11^. EMT is triggered through signals that cells receive from their environment, like the TGFB signal which is a potent driver of EMT^12^. As EMT is a diverse process, research that focuses on EMT must take this into consideration and must understand EMT on a more diverse palette. Monitoring EMT-induced changes on different levels, such as those of gene expression, phenotypical (microscopically observable) changes and functional disturbances, has become increasingly important^13^.

EMT is a spatial process, where several key factors change their localization. The most obvious examples of these EMT-induced localization changes include EMT-related transcription factors, which translocate from the cytoplasm to the nucleus to exert their transcriptional activity^14^. One specific regulatory protein of EMT is beta-catenin (CTNNB), which translocates between the plasma membrane and the nucleus^15^. Although the role of protein translocations during EMT is evident, yet there is no work that evaluates how the compartmentalization of a network model may create better model predictions and how to systematically create models that incorporate data on subcellular dynamics.

Representing intracellular signalling pathways as signalling networks has become a standard for the evaluation of cellular signalling processes in the last decades^16–18^. These networks can be easily translated to Boolean models and these models offer a quick and effective option to examine dynamic biological processes. Boolean models define a node’s state as active (TRUE/ON/one) or inactive (FALSE/OFF/zero) and convert biological rules into Boolean rules using the logical operators AND, OR and NOT. These models are simplified but are still very useful to qualitatively evaluate dynamic biological processes^19^.

EMT was previously characterized by several computational models^20^, but these often focus on very specific aspects of the transition^21^ or just on some core regulatory proteins^22^. An extensive attempt that incorporated a large number of proteins and characterized EMT on a system level was published by ref. ^23^. That model revealed the joint Sonic Hedgehog and Wnt pathway activation during TGFB-mediated EMT. This model consisted of 70 nodes and 135 edges, but using an attractor-preserving network simplification method it was reduced to a 19 node core network. In the current work, we present how the addition of compartment-specific information enriches the usefulness of a Boolean network model. For this purpose, we used the 19-node EMT model published in the paper of Steinway et al. as a benchmark and we compared our compartment-extended model to it. We refer to this 19-node EMT model of ref. ^23^ as the “original EMT model”.

Our work systematically incorporates protein translocation into a Boolean dynamic signalling model, thus it catalyses the understanding of protein translocation as a general and important aspect of cellular regulation. In the future, similar works will be useful to understand how protein translocations govern different cellular mechanisms and this will increase the understanding of how subcellular dynamics affect cellular behaviours. Extended models that use this compartmentalized approach will be useful to uncover novel biomarkers and to create more complex therapeutic targeting strategies.

In this work, we show the enrichment of translocating proteins in EMT and create a compartmentalized dynamic network model of EMT. Utilizing the potential of our compartmentalized model, we successfully prove that the addition of translocating proteins can result in a more concise decision-making system, by reducing the number of ambiguous attractors. More importantly, our model enables the analysis of compartment-specific protein functions, thus we were able to predict and validate (based on published experimental data) that both GSK3B and GLI compartment-specifically influence EMT.

## Results

### Enrichment of translocating proteins between signalling proteins

The Translocatome database contains the translocation probability (stored as Translocation Evidence Score) of approximately 13 thousand human proteins^5^. Based on the literature, translocating proteins are playing a pivotal role in cellular signalling and also in the EMT^14,24^. To prove this, we assessed the enrichment of translocating proteins between human signalling and EMT proteins. There are 13,066 human proteins in the Translocatome database, out of which 66% are non-translocating proteins, 25% are low-confidence and 9% are high-confidence translocating proteins. We assessed if the same distribution is observed between human signalling and EMT proteins. Signalling and EMT proteins were defined using specific Gene Ontology (GO)^25^ terms (see Methods for details).

**Fig. 1:**
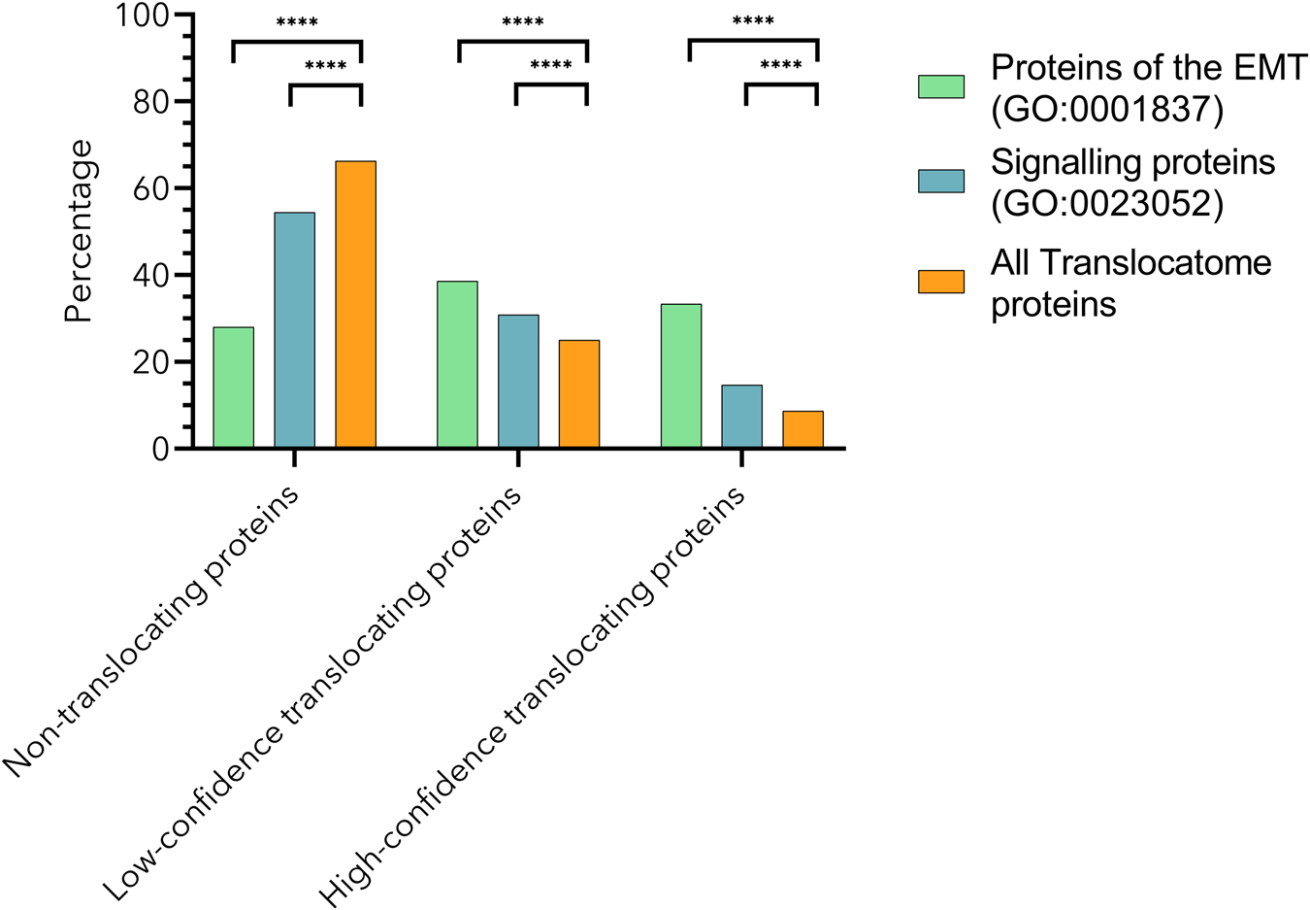
Translocating proteins are enriched between signalling and EMT proteins. On this figure, we can see the distribution of proteins according to their translocation probability in different datasets (Translocatome proteins, Signalling proteins and EMT proteins, see Methods for details). There were significantly more high-confidence translocating proteins between the Signalling (15%) and EMT (33%) proteins than between the Translocatome proteins (9%). We can observe the same significant difference in the case of low-confidence translocating proteins. The percentage of low-confidence translocating proteins is higher between Signalling (31%) and EMT proteins (39%) than between Translocatome proteins (25%). Oppositely in the case of non-translocating proteins there is a lower percentage of non-translocating proteins between signalling (54%) and EMT (28%) proteins than between Translocatome proteins (66%). For further details about the calculation of protein enrichment refer to the Methods section, and to understand the details of the Translocatome data please refer to its original publication^5^. ****p < 0.0001, Chi-square test.

We show that the observed distribution of translocating proteins differs in the case of signalling and EMT proteins, from what we would expect based on the Translocatome database (Fig. 1). Between signalling proteins, we found a higher percentage of translocating proteins (31% are low-confidence and 15% are high-confidence translocating proteins) and fewer non-translocating proteins (54%). Similarly, in the case of the proteins in connection with EMT, we also found a higher percentage of translocating proteins between the EMT proteins (39% are low-confidence and 33% are high-confidence translocating proteins) and the number of non-translocating proteins decreased (28%).

These observed discrepancies are significant (*p* < 0.0001), so we can conclude that translocating proteins become enriched in human signalling processes and in EMT (see details of this enrichment analysis in the Methods section). This observation also underlines the value of creating a compartmentalized model, enabling the possibility to represent protein translocation in a dynamic model.

### Creation of a compartmentalized EMT network

**Fig. 2:**
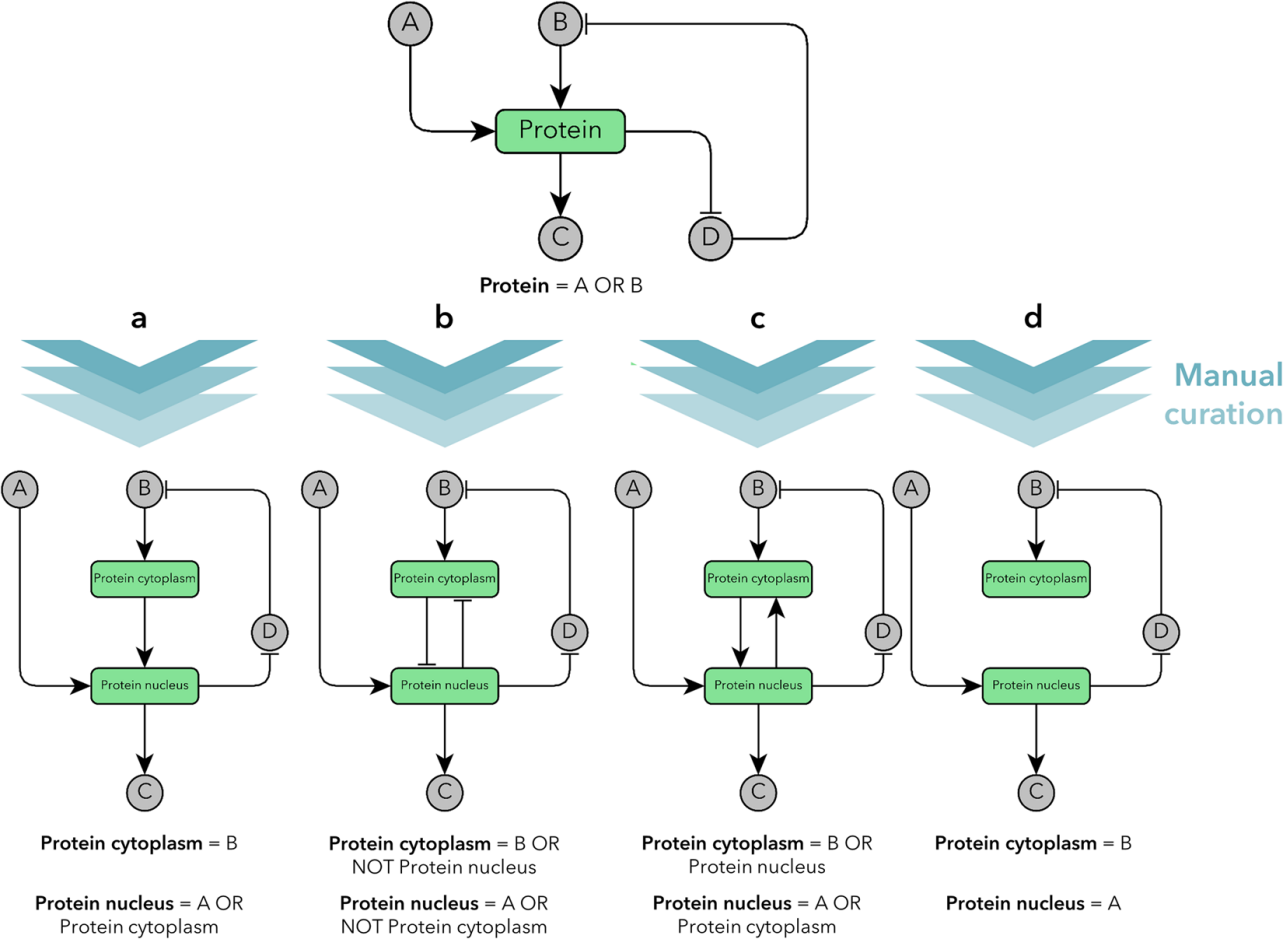
Creation of compartmentalized Boolean rules. Conventionally in the network representation of signalling processes nodes are individual proteins and edges (either inhibitory or activatory) are interactions between them, but their localization specificity is not considered. We take into consideration the compartment-specific functions of proteins so we can systematically add this information to Boolean models. After the careful revision of available literature data (manual curation) we created a compartmentalized Boolean network. In this hypothetical explanatory example we highlighted a “translocating protein” with green. **a** One possibility is that after e.g., phosphorylation by B the protein translocates to the nucleus and this does not directly affect the cytoplasmic pool of that protein. **b** Another possibility was already contained in the original EMT model (in the case of CTNNB), where the nodes mutually inhibit each other. **c** Some transcription factors can upregulate their own expression which can act as a positive feedback, **d** but it is also possible that both the cytoplasmic and the nuclear pools of a protein have their own regulatory interactions but they don’t affect each other directly. There are also other potential combinatorial possibilities (see Supplementary Fig. 7). During compartmentalization we always followed the logic of the available experimental data and tried to interpret into Boolean rules. As the nodes represent the functionality not the mass of proteins the simultaneous activation of both nodes does not negate the law of mass conservation (more on this topic in Supplementary Note 5).

After demonstrating the enrichment of translocating proteins in EMT we became interested in creating a network model of EMT where we can assess protein translocations from a network biology perspective. To model protein translocation, we created a compartmentalized network where we can represent each protein according to its subcellular localization. In this model, if a protein can translocate between 2 subcellular localizations, then it is divided into 2 nodes and each node has only the edges corresponding to that localization (Fig. 2). We call this process the compartmentalization of a network because during this process each protein’s regulation will be represented according to their subcellular locations. Since there is no universal solution to split one node into two compartmentalized nodes and there are several possible combinatorial scenarios it is important to capture the functionality of proteins. We always implemented the Boolean rules that best capture the biological behaviour of certain proteins, with a special focus on functionality and less on a universal methodology. This compartmentalization is crucial as many translocating proteins can have very different roles in different subcellular locations, e.g., there are translocating moonlighting proteins^26,27^.

In this work, our aim was to show the applicability and usefulness of the addition of compartment-specific information to a Boolean network model. To prove this, we used the 19-node EMT model published by ref. ^23^ as a benchmark and we compared our model to it. As mentioned earlier we refer to this model as the “original EMT model”.

The original EMT model had 19 nodes and 70 edges. The Translocatome database provided sufficient data to examine the translocation probability of these nodes. Out of the 19 nodes 14 nodes were predicted as high-confidence translocating proteins. After this initial signal that there are several important proteins that translocate during EMT, we carried out a thorough manual curation (see details in the Methods section), to validate these translocations, and to see if these translocations could be observed during EMT (summary of the manually curated information in Supplementary Data 1). If we could both validate the translocation itself, and its involvement in EMT, then we could create compartmentalized Boolean logic for that given protein.

In the compartmentalization phase, we created Boolean rules based on the literature, which was collected by manual curation. During this process, we reviewed 64 publications and created 10 compartmentalized nodes (see the list of these nodes and their Boolean rules in Supplementary Data 2) and thus by node-duplications extended the 19-node network into a 30 node-network (the NOTCH node was divided into 3 subcellular nodes, since it has validated localization and activity in the plasma membrane, in the cytosol and in the nucleus as well). After this, we had a model which was ready for further dynamic network analysis to understand the changes caused by the compartmentalization and to validate the model with the comparison to experimental results published earlier or after our predictions. The final model is defined in Supplementary Data 3 and we will refer to the node names defined in this Supplementary Data 3.

**Fig. 3:**
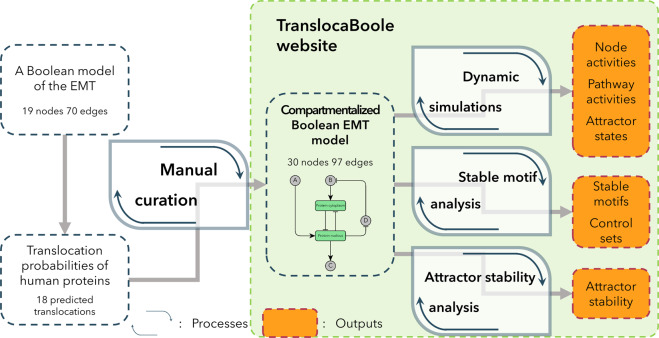
Workflow for the creation and evaluation of a compartmentalized Boolean model. Starting from previously published Boolean models of the EMT with the help of the Translocatome database^5^ it is possible to uncover translocating nodes that need to be compartmentalized. The careful revision of available literature i.e., manual curation, through the creation of new Boolean functions (based on the collected experimental data) enables the creation of a compartmentalized model. This is followed by dynamic simulation which could be analysed as different outputs on different levels, such as node or pathway activities or the identification of stable attractors of a system. Other analyses can identify the stable motifs and control sets of a model or calculate attractor stability measures. The website (https://translocaboole.linkgroup.hu/) enables users to modify our model and to automatically run simulations with our standard settings (25 iterations and 20,000 steps, every node is perturbed) and download the results.

This work can be generalized to a workflow shown in Fig. 3, where first the predicted translocations are identified in a previously published dynamic signalling network model, then these are manually validated and a compartmentalized network model is created according to the considerations shown in Fig. 2. Finally, computational simulations can be implemented on the compartmentalized network model and different outputs can be evaluated. The https://translocaboole.linkgroup.hu website provides a surface to edit compartmentalized Boolean rules and to rerun computational analysis (the simulation runs with the default settings of 25 iterations and 20,000 steps, every node is perturbed). Starting from the Boolean rules of our model users are able to change them. Once this is done, the server supporting the website automatically generates the required inputs needed for the dynamic simulations in the background, and users can download the final output files of these analyses (Fig. 3).

Users who need more features and modifications can also opt to download the input files and with those files, they can use the relevant codes available in our GitHub repository (https://github.com/deriteidavid/compartmentalized_EMT_Boolean_model_Mendik_et_al_2021) to rerun the analyses (for details see Supplementary Fig. 1). The website and GitHub tools make it possible to also use our workflow as a framework for similar future studies.

### Characteristics and dynamical repertoire of the compartmentalized EMT model

In the previous steps of our work, we created a compartmentalized signalling network that could easily be translated to a dynamic model based on the work of ref. ^23^. The resulting model is a discrete dynamic Boolean model that characterizes every node by a state variable (ON or OFF) corresponding to the node’s activity and a Boolean function that represents the node’s regulation. During the dynamic simulations, an asynchronous updating algorithm randomly selects a node and updates its state according to its Boolean function (more details in Methods). The arguments of the Boolean functions represent the transcriptional, post-transcriptional, translational, post-translational or localizational regulation of a certain node. The main predictive power of Boolean models of biological systems lies in their long-term convergent behaviour into stable states, which qualitatively correspond to real biological phenotypes^28,29^. The attractors of a Boolean model are such terminal stable states. The predictive value of a Boolean model of a biological system can be assessed by evaluating its attractors from a biological viewpoint. Moreover, it is also crucial to understand how the attractor states emerge from the dynamical landscape of the model. This understanding can enhance further validation of the model and give insight into control and finding potential therapeutic targets^30,31^.

**Fig. 4:**
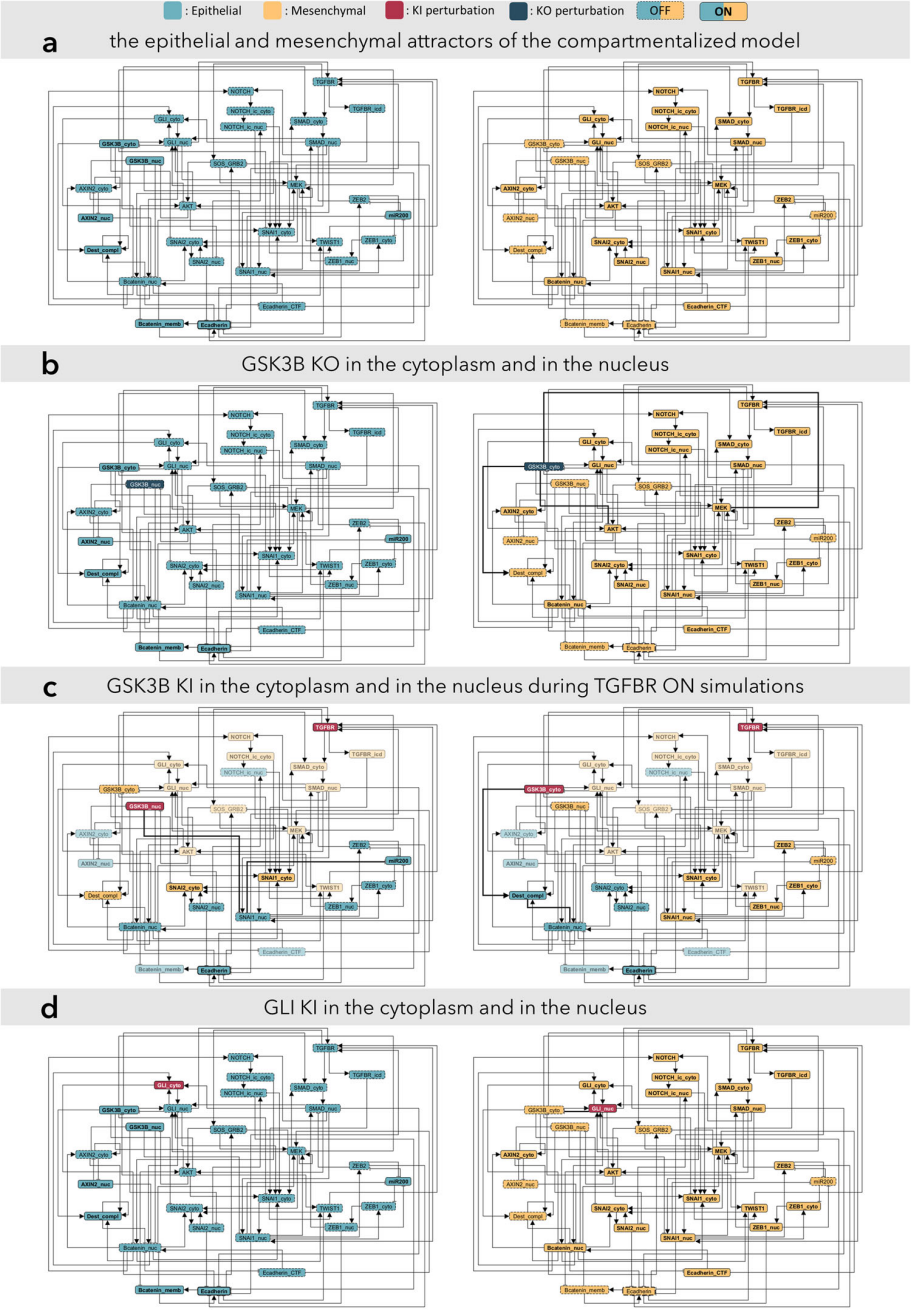
Attractors of the compartmentalized model showing localization-specific functions of GSK3B and GLI. **a** The attractors corresponding to the epithelial and mesenchymal state can be seen on the left and right respectively. During the dynamic simulations, perturbations were introduced to the initial epithelial state. **b** In case of the TGFBR OFF simulations the cytoplasmic KO of GSK3B resulted in EMT, while the nuclear perturbation had no effect. The signal from GSK3B_cyto KO propagated through the loss of the Dest_compl and the activation of AKT and MEK. **c** During TGFBR ON simulations both the KI of the cytoplasmic and nuclear node of GSK3B inhibited the TGFB mediated EMT. The nuclear perturbation had a greater inhibitory effect by preventing the loss of miR200 and consequently the activation of ZEB1 and ZEB2. The nuclear perturbation also led to the inhibition of SNAI2_nuc and Bcatenin_nuc, despite the loss of the Dest_compl and the activation of SNAI2_cyto compared to the cytoplasmic perturbation. GSK3B_cyto functions both to stabilize the epithelial state and to inhibit TGFB mediated EMT, whereas GSK3B_nuc functions specifically to inhibit TGFB mediated EMT **d** TGFBR OFF simulations show that the nuclear perturbation of GLI led to EMT, while the cytoplasmic perturbation alone was insufficient to destabilize the epithelial state. As part of a transcription factor family, the GLI proteins exert their main function in the nucleus and if sequestered in the cytoplasm, GLI2 gets truncated into a repressor form that further decreases transcriptional activity. The compartmentalized model reproduced these localization-specific functions.

Using a state-of-the-art method^32^ we mapped out the dynamical repertoire of our new model in the form of a succession of stable motif stabilizations. Stable motifs are generalized positive feedback loops (also known as maximal trap spaces) which represent sets of self-sustaining node-states, which once activated permanently lock into those node-states^33^. Different successive combinations of stable motifs determine the steady state attractors of the system. The new compartmentalized EMT model has seven attractors summarized in Supplementary Table 1. The two attractors most relevant in our case are the ones corresponding to the epithelial (E) and mesenchymal (M) states (Fig. 4a). The E and M attractors are polar opposite states, differing in every node state (with the exception of SOS_GRB2 which is 0 in both attractors). The remaining 5 attractors are intermediary states, which we call hybrid attractors. Hybrid states can emerge during incomplete or partial EMT processes^24^. The emergence of the hybrid attractors of our model can be explained with three specific stable motifs, made up of only 5 nodes in total, as discussed in Supplementary Note 1 (see also Supplementary Fig. 2 and Supplementary Table 2).

Furthermore, based on the method of refs. ^32,34^ we compared the control sets of our model with the original EMT model. A control set of an attractor is a given group of nodes, which, when forced to the corresponding state, drive the whole system into a specific attractor^35^. As the data in Supplementary Data 4 show the simplest control set of the epithelial state is a smaller set of nodes (5 nodes, see Supplementary Data 4, Epithelial control sets of the Compartmentalized model No. 1) compared to the original EMT model (6 nodes, see Supplementary Data 4, Epithelial control sets of the Original model No. 1), thus our model reveals that forcing the network into the epithelial state is more feasible if we use compartment-specific perturbations.

We also found that in the presence of noise (introduced to our model as update errors) the mesenchymal attractor is by far the most stable and thus has the highest stationary probability from all states (for details see Supplementary Note 2). This results from the fact that this original EMT model focuses on the transition from epithelial to mesenchymal state and thus nodes and interactions driving towards the mesenchymal states are overrepresented.

It is also worth mentioning that although the overall stability profile of attractors still overwhelmingly favours the mesenchymal state, the balance is somewhat improved towards epithelial state as compared to the original EMT model of ref. ^23^ (see Supplementary Table 3).

### Comparison of the original and compartmentalized model

As mentioned above our simulations showed that the compartmentalized model has both a stable epithelial and mesenchymal state. Our compartmentalized model was validated by showing that it could reproduce key experimental outcomes (detailed in Supplementary Table 4) that had been used for the validation of the original EMT model.

For additional comparison and analysis of the two models, we used two simulation setups: one we call single node TGFBR node OFF (simply referred to as TGFBR ON or OFF), the other TGFBR ON simulations. In both setups the network started from the epithelial state and was perturbed by permanently setting a node’s state to ON or OFF, thus overriding its Boolean rule. These perturbations were similar to wet-lab knock-out (KO) or knock-in (KI) experiments, but importantly, our perturbations were always compartment-specific. This resulted in scenarios when a protein’s functionality was maintained in one subcellular compartment and inhibited in another. During the TGFBR ON simulations the epithelial initial state was altered by setting the TGFBR node’s state to ON and an additional KI/KO perturbation was also introduced to the network. This represented the biological scenario of an active TGFB signal which is a potent driver of EMT. In the latter case, we observed if a node’s perturbation can block TGFB driven EMT.

Comparison of TGFBR OFF simulations showed differences between the original and our model in two cases: NOTCH or MEK KI led to EMT in the original but not in the compartmentalized model. In the case of NOTCH activation, our model better simulated the experimental results showing that Neurogenic locus notch homolog protein 1 (NOTCH) activation alone without the induction of TGFB was not sufficient to induce EMT^36,37^ (Supplementary Figs. 3 and 4). This result stemmed from the fact that in our model the activation of Zinc finger protein SNAI1 (SNAI1) is captured more complexly, and NOTCH alone could not activate this key transcription factor of EMT.

In the case of MEK activation, there was a more complex situation. The activation of the MEK node did not lead to a mesenchymal phenotype in the compartmentalized model, whereas it did in the original EMT network (Supplementary Figs. 3 and 4). There is available experimental validation for both outcomes, as experiments have shown that MEK/ERK pathway activation alone did not, while a combined TGFB and MEK/ERK pathway activation did lead to a perfect EMT phenotype in a lung adenocarcinoma cell line^38^. However, in intestinal epithelial cells overexpression of a constitutively active form of MEK1 activation was sufficient to induce EMT^39^. Moreover, in the case of TGFBR ON simulations only the compartmentalized model showed correctly that MEK inhibition could prevent TGFB induced EMT^40^ (Supplementary Fig. 5).

### Compartmentalized functions of GSK3B and GLI

The division of translocating nodes by compartments allows for the exploration of compartment-specific functions, providing the major advantage of our method. In TGFBR OFF simulations GSK3B KO in the original EMT model led to a mesenchymal phenotype, which matched the outcome of GSK3B_cyto KO in the compartmentalized model. Experimental evidence underlines this outcome as the inhibition of cytoplasmic GSK3B by LiCl led to EMT in ovarian adenocarcinoma^41^. The compartmentalized model uncovered that GSK3B_nuc KO did not lead to EMT (Fig. 4b). Because GSK3B localizes mainly to the cytoplasm^41,42^ and translocates to the nucleus during EMT^43^, it is expected that in the epithelial state GSK3B_nuc KO does not have an effect.

Furthermore, in the case of TGFBR ON simulations, we could predict other compartment-specific functions of the GSK3B kinase. Our results showed that the KI of GSK3B both in the cytoplasm (GSK3B_cyto) and in the nucleus (GSK3B_nuc) acted as a repressor of EMT, but this inhibitory effect was stronger in the nucleus (Fig. 4c). In accordance with that, experimental results proved that the inhibition of the PI3K/AKT pathway suppressed EMT through the induction of GSK3B in hepatocellular carcinoma (HCC)^43^. The more robust inhibition in the nucleus is a consequence of the fact, that nuclear translocation of GSK3B prevents EMT through the downregulation of SNAIL transcription factor^43^ and that nuclear GSK3B is highly active relative to its cytoplasmic counterpart^44^.

Another node that showed compartment-specific functions is the GLI node. GLI KI resulted in EMT in the original model just as GLI_nuc KI led to EMT in the compartmentalized model. Importantly, it has been reported in the literature that independent GLI activation can induce EMT^45^. Additionally, the compartmentalized model shows that GLI_cyto KI did not result in EMT (Fig. 4d). This is due to the fact that in the absence of an upstream signal cytoplasmic GLI2 gets truncated into a transcriptional repressor form which inhibits GLI-induced gene transcription^46^ and there is a simultaneous cytoplasmic sequestration of GLI1^47^. Overall, we have shown that our compartmentalized model compared to the original could provide justifiable results on the subcellular regulation and compartmentalized functions of different kinds of proteins like the GSK3B kinase and the transcription factor family GLI.

### Signalling pathway analysis uncovers pathway crosstalk

To understand how different signalling pathways activate during the EMT we created a method to assess not only node level but signalling pathway activities as well (see the details of this in the methods section). We monitored the activity of the most significant signalling pathways that lead EMT^14^. These pathways are:

- TGF-β SMAD-dependent signalling
- TGF-β SMAD-independent signalling
- receptor tyrosine kinase (RTK) signalling
- Wnt signalling
- Notch signalling
- Hedgehog signalling
- Hypoxia signalling.

**Fig. 5:**
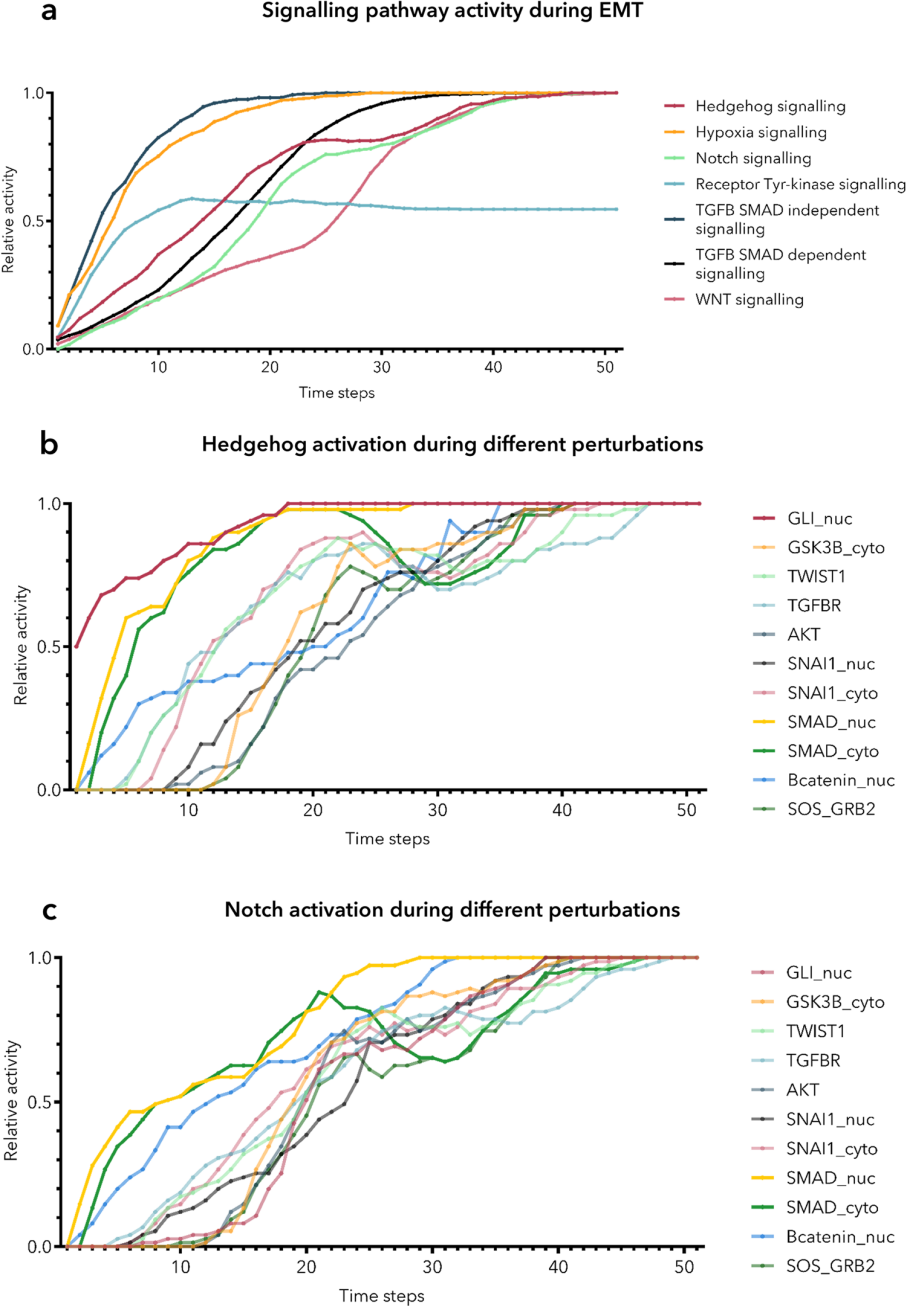
Signalling pathway activities during EMT. **a** We plotted the activity of the main pathways during EMT on this panel. There are 11 single node perturbations that led to EMT, here we show the average of the pathway activities that can be observed during these 11 perturbations. We can see that there was a difference in the kinetics of the activation of different pathways. Importantly, we could still observe that TGFB activation is accompanied by the activation of the Hedgehog and Wnt pathways as captured by the original EMT model^23^. **b** On this panel we show the activation of the Hedgehog signalling pathway for different perturbations. Each line symbolizes one specific perturbation which led to EMT and thus the activation of the Hedgehog pathway. We note here that the perturbation of the TGFB pathway member SMAD proteins (yellow and green) resulted in a quicker and more robust activation of the Hedgehog pathway. The most robust activation could be observed when we activated the nuclear pool of GLI proteins, with the perturbation of the GLI_nuc node (brown). **c** Here, we represent the activation of the Notch signalling pathway for different perturbations similarly as we showed it in the case of Hedgehog signalling on panel b. Here the activation of SMAD proteins resulted in a quick and robust activation of the Notch pathway which underlines the crosstalk between these pathways.

This signalling pathway-level analysis confirmed that WNT and Hedgehog pathways are jointly activated during EMT as it was highlighted in the case of the original publication^23,48^ (Fig. 5a), but also captured the activation of other important pathways (RTK, Notch and Hypoxia signalling). Moreover, the activatory perturbation of SMAD proteins resulted in a quicker activation of the Hedgehog signalling pathway highlighting validated crosstalks^49,50^ between the TGFB and Hedgehog signalling pathways (Fig. 5b). Further analysis also captured the synergic functions between TGFB and Notch signalling^51,52^, as the activation of TGFB pathway-member SMAD proteins led to quicker activation of the Notch pathway (Fig. 5c).

## Discussion

In this work, we presented the importance of translocating proteins in the signalling of the EMT and created a framework of how to create a compartmentalized Boolean model using a previously published reference work^23^. Using this model, we simulated the EMT and presented key outcomes which can be considered as emerging properties of this new model. These new outcomes described experimental results better than the original EMT model due to our model’s capability of better capturing compartment-specific functions of proteins.

It is known that a large proportion of proteins may localize to multiple compartments inside a cell^10^. This subcellular organization and the underlying dynamics are essential for physiological functions of human cells^53^. We have numerically proven that translocating proteins are overrepresented between human proteins in the subset of human signalling and human EMT associated proteins (Fig. 1). We then created a framework which enables the incorporation of translocating proteins into cellular signalling models. We believe that the addition of translocating proteins to other models will greatly enhance in silico models and future studies.

First, we have presented that our model is able to recapitulate the results of the original EMT model. Our model maintains a stable epithelial and mesenchymal steady state (Fig. 4a), and certain perturbations (e.g.,: TGFB signal) can lead to a mesenchymal phenotype. It is important to note that this model simulates the transition from the epithelial state to the mesenchymal state so the mesenchymal state is the most robust attractor (Supplementary Table 1), and the inverse process of MET cannot be studied in this model (due to the fact that the regulations of this latter process are not involved in our model). With the above in mind, we can still conclude that our model produces biologically valid stable attractors with a slight enhancement in the stability of the epithelial attractor (which was very vulnerable to perturbations in the original model. See Supplementary Note 2 for details).

Based on the control sets of our model (Supplementary Data 4) we can also see, that the compartmentalization of a model reveals new control possibilities as expected. This is recapitulated by the fact, that our model reveals nontrivial compartmentalized node interventions. Hypothetically based on our model the control of the epithelial state can be reached by knocking out nuclear and cytoplasmic pools of GLI and AXIN2 proteins, respectively. In case of these compartment-specific perturbations there is no need to additionally knock out SNAI2 as in the case of the original EMT model (see Supplementary Data 4 Epithelial control sets No. 1 of the Compartmentalized and the Original model). Perturbations based on control methods may signal only in vitro intervention points, thus the control sets identified in compartmentalized models should be later validated through wet-lab experiments.

In comparison with other models, we must emphasize that there are not many system level models of the EMT^20^. Kinetic and ordinary differential equation (ODE) models were shown to be useful in deciphering certain aspects of EMT, but they focus on a very limited number of nodes. To highlight some of such available models, one focused on a general minimal dynamic model and numerically described EMT based on among others the analysis of the phase diagram^22^. Another specific model addressed the shuttling of CTNNB and its antagonists^21^, and a third one focused on the bistable switches underlying EMT^54^. Although these models are useful in the quantification of dynamics but have a high computational demand. Thus, these models cannot be scaled up to model processes on a system level and they cannot be used to understand system level complexity and to plan system level interventions. The model of ref. ^55^ also builds on the Boolean model of ref. ^23^ but uses a slightly modified modelling approach and incorporates the LIF/KLF4 pathway to the model. Their model showed a more symmetric and more diverse representation of the attractor states involving a multitude of metastable hybrid phenotypic states. To test the robustness of our results we included the LIF/KLF4 pathway to our model. In this modified model we still observed the same compartment-specific functions (Supplementary Note 3), and no notable difference in the attractor landscape. This gives an important additional proof for the robustness of our method. Throughout this paper, we extensively compared our model with the Boolean model of ref. ^23^, which models EMT systematically, and our model succeeded the original EMT model in predicting compartment-specific functions.

The most important aspects of this study are those which highlight the compartment-specific functions of proteins. One of our most significant results is that GSK3B compartment-specifically inhibited EMT. We’ve predicted that nuclear activation of GSK3B can interfere with EMT and it prevents the activation of key factors of the mesenchymal state (Fig. 4c). In their paper. Lee et al.^43^ created an experimental constellation in which exactly this phenomenon was examined. It is important to note that their paper was published after our initial results, so our results could be considered as computational predictions that are validated by this very recent experimental study. Lee et al.^43^ claimed that enhancing the nuclear translocation of GSK3B i.e., the activation of GSK3B in the nucleus suppresses EMT in HCC cells^43^.

Another result showed that GLI transcription family members compartment-specifically induced EMT (Fig. 4d). Our findings coincide with other studies showing that GLI1 aberrantly activates in DNA damage and carcinogenesis^56^ and it activates EMT due to its transcriptional activity mainly through SNAI1^56^. There are promising therapeutic targets among the SHH pathway members. Both GLI antagonists (GANTs) and Arsenic trioxide (ATO) (which also inhibits GLI functions) have been shown effective^57^. Our simulations correctly identified GLI as an important factor during EMT, which is highlighted by the above examples. Further simulations that incorporate the actors of the SHH pathway complexly, could shed light on more impactful interventions that target GLI proteins.

Lately EMT is described as a multifaceted and often reversible process. To define EMT in its enormous complexity and plasticity in diverse developmental and pathological settings, studies must focus on complex molecular markers and changes in cellular properties simultaneously^13^. With this mindset we focused on multiple aspects of EMT during this study. We analysed attractors in their entirety and did not rely solely on some specific markers (e.g., loss of E-cadherin (CDH1) alone, or a dedicated EMT node). We also analysed signalling pathway activities (Fig. 5) which represented EMT more on a spectrum rather than just a binary outcome. Furthermore, we aimed to create a functional readout based on GO^25^ terms, but this has limited usability as GO terms currently cannot capture the compartment-specific traits of proteins (see Supplementary Note 4 and Supplementary Fig. 6 for more on this matter).

In summary, we presented the enrichment of translocating proteins in EMT and to model their role we created a compartmentalized Boolean dynamic model of the EMT. This model was used to prove that translocating proteins, namely GSK3B and GLI proteins, can alter the fate of EMT in a compartment-specific manner. Our study also serves as a framework for future studies thanks to the algorithmic procedures available on the https://translocaboole.linkgroup.hu website and in the GitHub repository of this project. The results of our model underline that proteins should be modelled in their specific location with compartment-specific functionality in order to maintain their physiological cellular behaviours. Also, the understanding of subcellular dynamics and the utilization of similar frameworks as ours in more complex networks will help uncover new biomarkers and therapeutic targeting strategies.

## Methods

### Enrichment analysis of translocating proteins

To validate the enrichment of translocating proteins in human signalling and specifically in EMT we assessed the enrichment of translocating proteins as detailed below. The only available proteome level database of human translocating proteins is the Translocatome database, containing 13,066 human proteins. The database characterizes each protein with a Translocation Evidence Score (reflecting the translocation probability of that given protein). We found that in the Translocatome database 66.28% of the proteins are non-translocating proteins, 25.04% are low-confidence translocating proteins and 8.68% are high-confidence translocating proteins.

We used the Gene Ontology (GO)^25^ database to identify the proteins, which take part in human signalling processes. To achieve this, we downloaded the list of proteins annotated with the GO term “signalling (GO:0023052)”. There were 6867 proteins annotated with this GO term and the translocation probability data (from the Translocatome database) was available in the case of 4991 proteins.

Similarly, we used the GO database to identify the proteins in connection with EMT. To achieve this, we downloaded the list of proteins annotated with the GO term “epithelial to mesenchymal transition (GO:0001837)”. There were 156 proteins annotated with this GO term and the translocation probability data (from the Translocatome database) was available in the case of 133 proteins.

In order to validate that the observed distribution of translocating proteins—in the case of signalling and EMT proteins – indeed significantly differs from the expected, we conducted Chi-square tests where we validated the observed datasets against the Translocatome database. In both cases, we found that the observed distributions significantly differ from the Translocatome database with a *P* value of smaller than 0.0001.

### Manual curation

We conducted manual curation of translocating EMT proteins guided by translocation probability predictions of the Translocatome database^5^ that classified 13,066 human proteins into three groups: high- and low-confidence translocating and non-translocating proteins. We utilized the PubMed and Google Scholar search engines to find the relevant scientific publications for protein-translocation in EMT. It consisted of two phases: the selection of translocating proteins of the 19-node network and the exploration of the regulatory relations of these proteins.

In the selection-phase, we searched for the evidence of proteins translocating during the signalling of EMT to assess how extensive the literature was on the translocation of these proteins. In the exploration-phase, we gathered the necessary information for the system-level incorporation and Boolean rule creation of validated translocations into the model. Similarly to the methodology that was used in the manual curation of the Translocatome database^5^ the following preferences/conditions were set for the refined keyword searches:

- selection-phase:
  ○ Word combinations in quotation marks and “title” or “title/abstract” field-restrictions were used to refine keyword searches.
  ○ The publication had to be peer-reviewed and had to connect the translocation of a human protein to EMT.
  ○ The described translocation had to meet the requirements of the definition established in ref. ^5^.
  ○ Recent publications (last 5 years) were preferred.
- exploration-phase:
  ○ Experimental evidence from hepatocellular carcinoma (HCC) cell lines were preferred otherwise other human carcinoma cell lines were preferred.
  ○ Publications exploring TGFB mediated EMT were preferred
  ○ Some publications were tracked down from the references or the “cited by” page of review publications or relevant experimental publications not quite congruous with our conditions.
  ○ Conditions/preferences of the selection-phase were also applied here.

In the selection-phase, we focused on finding evidence in the literature for the fact of a protein of the 19-node network translocating during EMT. Based on 64 publications we collected data about 21 proteins (see Supplementary Data 1) in the following topics:

1. name set, gene name and UniProt^58^ accession number and link,
2. PubMed ID(s) and link(s) to peer-reviewed article(s) describing the experimental evidence of translocation,
3. initial and target localizations of the translocating protein,
4. interacting partners and biological functions (both in the initial and target compartments),
5. translocation mechanism,
6. the used detection method,
7. protein structural information on translocation mechanism,
8. disease group, exact disease involved and pathological role,
9. signalling pathways affected.

In the exploration-phase, we first searched for a review article of the molecular signalling mechanisms of EMT^14^ that could show us the well-established translocation events in the signalling of EMT. We then gathered information on the mechanism of the translocations (e.g., its regulation or dynamics) and the compartmentalized protein-protein interactions of the validated translocating proteins. We prioritized the review of publications that described interactions of a translocating protein with other proteins that were in the original network. For a detailed transcript of the manually curated information please refer to Supplementary Data 1.

### Boolean network modelling and dynamic simulations

For the modelling of the compartmentalized EMT network inferred from the manually curated information, we utilized the Boolean framework described by ref. ^23^. In this every node is characterised by one of two qualitative states: ON (also referred to as TRUE or 1) or OFF (FALSE or 0) meaning above or below threshold activity/abundance, respectively. The state of a node is determined by its Boolean rule, which is a Boolean function that expresses the upstream regulatory relationships of the node. The Boolean rules of the compartmentalized model can be seen in Supplementary Data 2.

We updated the BooleanNet software package^19^ for the dynamic simulations of the model (see Data/Code availability section for the GitHub repository). The initial state of the simulations was the epithelial state. If perturbed by setting a node’s state to a fixed OFF or ON state, the system may have converged into an altered attractor (corresponding to the steady mesenchymal state or a partial epithelial/mesenchymal state). Due to the stochastic nature of the unranked asynchronous update mode the outcome of a perturbation and the order of node-state updates could differ between simulations, so we performed 100 iterations of TGFBR OFF and 25 iterations of TGFBR ON simulations to assess these differences (there was no difference in the outcome of TGFBR simulations with 25 or 100 iterations). For validation, we compared the results of the perturbation analysis to experimental evidence found in the literature.

### Signalling pathway analysis

In order to observe the activation of different signalling pathways during the course of our simulation we created a method to observe signalling pathway activity. Based on the work of Gonzalez et al.^14^ we mapped each node in our model to one of six major pathways contributing to EMT and identified the node’s Boolean state which corresponds to signalling activity. This data is summarized in Supplementary Table 5. With this data, we were able to compute the activity of each of the six tracked signalling pathways in each timestep during a simulation. The activity of a signalling pathway is the average activity of the nodes which belong to that pathway.

### Gene Ontology based functional analysis

Similarly, to the signalling pathway analysis, we aimed to create a functional analysis of our model as well. The Gene Ontology (GO) database^25^ annotates all the human proteins with the biological processes connected to them. We monitored the activity changes of nodes during EMT and with the simultaneous utilization of the GO data we were able to translate this node activity into the activity of biological processes. These mapped biological processes correspond to the core changes of the EMT:^13^

- cytoskeleton organization (GO:0007010)
- establishment or maintenance of epithelial cell apical/basal polarity (GO:0045197)
- epithelial cell-cell adhesion (GO:0090136)
- cell-matrix adhesion (GO:0007160)
- cell motility (GO:0048870).

Besides these, we also monitored the activity of the GO term “epithelial to mesenchymal transition (GO:0001837)”.

In order to translate node activities to process activities we identified which nodes’ activity represent which process’ activity, then the activity of a process is the average activity of the underlying nodes’ activity.

### Attractor stability analysis

We were interested in how the stability of attractors in our compartmentalized EMT model compares to the 19 node Steinway et al.^23^ model. To measure this, we conducted a numerical experiment on both models: we introduced an update noise, which returned the wrong state for every update with probability *p*_error = 0.001. This made it possible for the system to leave any attractors. Next, we simulated the long-term time-evolution (106 general asynchronous update steps) of the model initialized from one of the attractors and monitored how much time it spends in each visited state. We did this for every attractor and summarized the visitation probabilities for each attractor.

## Supplementary information

Supplementary Material

Supplementary Data S1: Collection of manually curated information used to compartmentalize the network

Supplementary Data S2: Boolean rules of the original EMT network and the compartmentalized network

Supplementary Data S3: Node definition of the compartmentalized model

Supplementary Data S4: Epithelial and mesenchymal control sets of the compartmentalized and the original model

## Data Availability

We made all relevant data available in the Supplementary Information, the Boolean model is defined in Supplementary Data 3 which is the most minimal dataset to reproduce our results. The codes used during the project are available in the GitHub repository: https://github.com/deriteidavid/compartmentalized_EMT_Boolean_model_Mendik_et_al_2021. In case of any further inquiry please contact the corresponding author.

## Code availability

The relevant codes used for data analysis and simulations are available in the GitHub repository: https://github.com/deriteidavid/compartmentalized_EMT_Boolean_model_Mendik_et_al_2021.
