# Supplementary material for: Translocating proteins compartment-specifically alter the fate of epithelial-mesenchymal transition in a compartmentalized Boolean network model
Source: NPJ Syst Biol Appl. 2022 Jun 9;8:19. doi: 10.1038/s41540-022-00228-7 (PMC9184490; doi:10.1038/s41540-022-00228-7)
Supplement: Supplementary file 1 — Supplementary Material [file 41540_2022_228_MOESM1_ESM.pdf]

Supplementary Information

Translocating proteins compartment-specifically alter the fate of epithelial-mesenchymal transition in a compartmentalized Boolean network model

Péter Mendik[1#], Márk Kerestély[1#], Sebestyén Kamp[2], Dávid Deritei[1], Nina Kunšič[1], Zsolt Vassy[1], Péter Csermely[1], Dániel V. Veres[1,2]

[1]*Department of Molecular Biology, Institute of Biochemistry and Molecular Biology, Semmelweis University, Budapest, Hungary*

[2]*Turbine Ltd. Budapest, Hungary*

Corresponding Author: D.V.V. veres.daniel1@med.semmelweis-univ.hu

Corresponding authors' address:

Semmelweis University

Department of Molecular Biology

H-1428 Budapest, P.O.Box 2

FAX: +361-266-3802

phone: +361-459-1500, extn.: 60130

www.linkgroup.hu

email: veres.daniel1@med.semmelweis-univ.hu

[#]The authors wish it to be known, that the first two authors should be regarded as Joint First Authors.

Table of content

Supplementary Notes 3
Supplementary Note 1: Three stable motifs explain the emergence of hybrid attractors 3
Supplementary Note 2: Stability of attractors 5
Supplementary Note 3: Effects of the involvement of the LIF/KLF4 pathway on our compartmentalized model 6
Supplementary Note 4: Constraints of the Gene Ontology-based functional characterization of our compartmentalized model 6
Supplementary Note 5: Correlation of node compartmentalization with protein mass and function.. 7
Supplementary Figures 9
Supplementary Figure 1: Algorithmic operations and program modules of our workflow........ 9
Supplementary Figure 2: Stable motif succession diagram of the new compartmentalized model.. 11
Supplementary Figure 3: Stable states of the original EMT model after single node perturbations.. 12
Supplementary Figure 4: Stable states of the compartmentalized EMT model after single node perturbations.. 14
Supplementary Figure 5: Uncovering the effect of MEK KO with TGFBR ON simulations.. 16
Supplementary Figure 6: Activity of 5 GO biological processes during EMT......................... 17
Supplementary Figure 7: All possible node compartmentalization scenarios 18
Supplementary Tables 19
Supplementary Table 1: Attractors of the compartmentalized model....................................... 19
Supplementary Table 2: List of stable and conditionally stable motifs in the compartmentalized model 21
Supplementary Table 3: Attractor stability in case of the epithelial and mesenchymal attractors in the compartmentalized and in the original EMT model.. 22
Supplementary Table 4: Experimental outcomes reproduced by the compartmentalized and the original EMT model 23
Supplementary Table 5: Nodes annotated to signalling pathways.. 24
Supplementary References 25

Supplementary Notes

Supplementary Note 1: Three stable motifs explain the emergence of hybrid attractors

We analysed our compartmentalized Boolean EMT model from the perspective of stable motif succession, stabilization and their role in locking in the dynamics into different attractors. Stable motifs (SMs) are generalized positive feedback loops, which represent sets of self-sustaining node-states, which once activated permanently lock into those node-states[1]. A more generalized form of stable motifs are conditionally stable motifs (CSMs), whose self-sustaining quality is contingent on external node states[2]. If the conditions of a CSM are locked in (e.g. in an upstream stable motif) they act as stable motif themselves, since their conditions are permanently satisfied. Because of this, and for the sake of brevity, we will generally refer to CSMs as stable motifs, only highlighting their conditional character if it is relevant.

Stable motifs and their successive stabilization determine the different irreversible pathways that will end up in attractors. Moreover, as we will see, stable motifs are key in understanding the emergence of the different attractors, including the hybrid states.

Supplementary Table 2 lists the stable motifs of the model. Supplementary Figure 2 represents the stable motif succession diagram of the model. The topmost nodes on the figure represent the stable motifs, which can lock in without any external condition. Arrows represent possible successive stabilizations of stable and conditionally stable motifs. The nodes at the bottom of the figure represent the attractors.

Attractors are combinations of consistent stable motifs. Two stable motifs are consistent with each other if they do not contain contradicting states of the same node. A single stable motif can be part of multiple attractors. On Supplementary Figure 2 we coloured the stable motifs that are consistent with the epithelial attractor (E) with blue. Stable motifs highlighted in orange are inconsistent with *all* epithelial (blue) motifs and they all inexorably lead to the mesenchymal attractor. Most important, however, in understanding the emergence of the hybrid attractors are the stable motifs highlighted with green (2,4,13). These stable motifs are consistent with all the mesenchymal motifs (orange) but are inconsistent with only some of the epithelial motifs (blue). This means that they

can simultaneously stabilize with the consistent epithelial motifs and create attractors that are neither fully epithelial nor fully mesenchymal. For example, following the stabilization going from the top from SM with the label 5 to SM 14 the system has a choice: it can next stabilize SM 2 (Bcatenin_memb= 0, Ecadherin= 0) or SM 12 (Bcatenin_memb=1, Ecadherin=1). Neither SM 2 or SM 12 are inconsistent with the upstream motifs (SM 14 and SM 5) but they are inconsistent with each other, thus they generate (at least) two different attractors. The possible combinations of the hybrid motifs with the epithelial motifs allow for the creation of the five hybrid attractors, beside the E and M states. This insight is crucial for further future validation of the model. For instance if any of the hybrid attractors are found to be biologically implausible, the primary target of our inquiry into removing that attractor would be in re-evaluating the rules making up the hybrid motifs. An example of this procedure is already implicitly implemented in this model, as compared to the 19 node version of the EMT model from Steinway et al.[3] In that model SNAI2=1 formed an additional hybrid motif, leading to multiple extra hybrid attractors. Due to the compartmentalization implemented in our compartmentalized EMT model, neither the nuclear nor the cytoplasmic version of SNAI2 forms a hybrid motif, thus making the predictions of the current model more parsimonious.

In the case when hybrid attractors are biologically plausible, but represent unhealthy states, understanding the possible bifurcations of the system from the healthy path to an unhealthy one (see the example above) can be critical in designing successful interventions.

On Supplementary Figure 2 all the stabilization paths that lead to the epithelial attractor are highlighted in blue. Every other path leads to either a hybrid or the mesenchymal attractor.

The stable motif analysis was conducted with the methodology described by Rozum et al.[4] with the software package available at: https://github.com/jcrozum/StableMotifs

Supplementary Note 2: Stability of attractors

The results of the attractor stability analysis (summarized in Supplementary Table 3) show that the mesenchymal attractor is by far the most dominant attractor in both models (over 94% probability). In the case of our compartmentalized EMT model the mesenchymal state is marginally less stable and the epithelial attractor has a fourfold increase in probability. Nonetheless the contrast between the two extreme states is still 4 orders of magnitude.

This contrast in stability is further emphasized by the fact that the (condition-free) stable motifs of the epithelial attractor, 5 and 6, are much more complex, and thus more difficult to stabilize as compared to the stable motifs of the mesenchymal attractor (0,1,2 and 4). Due to this, non-locked states are much more likely to be locked into the mesenchymal attractor by noise or targeted interventions.

We confirmed this conclusion using stable motif control theory[4,5], which finds sets of interventions which can drive the system from any state into a desired attractor. The control sets of the mesenchymal attractor consist of single node interventions while the control sets of the epithelial attractor consist of sets of at least five simultaneous interventions. We note here that the minimum five simultaneous interventions in the case of our compartmentalized model is an improvement compared to the control sets of minimum 7 interventions in the Steinway et al.[3] model.

Supplementary Note 3: Effects of the involvement of the LIF/KLF4 pathway on our compartmentalized model

To test the robustness of our results we examined our results using a similar modelling approach published by Font-Clos et al.[6] In their work Font-Clos et al.[6] enriched the original EMT model of Steinway et al.[3] with the LIF/KLF4 pathway. They have added 4 nodes and 9 edges to the system and removed 1 edge from the network. In our work we used the reduced network of Steinway et al.[3] with only 19 nodes, thus we needed to simplify the LIF/KLF4 pathway similarly. This resulted in the addition of the KLF4 node (which is inhibited by ZEB2 and activates E-cadherin) to this reduced network.

Rerunning our simulations after the addition of the KLF4 node as discussed above showed, that the compartmentalized functions of GSK3B and GLI are still present, and there is no notable difference in the control sets or the attractor landscape (and the probability of different attractor states). These gave an important, additional proof to the robustness of our method. Notably the knock-in of the GSK3B_nuc node resulted in a partial MET as the E-cadherin node became activated (even during TGFBR activation). This indicates that the inclusion of the LIF/KLF4 pathway is important in larger-scale studies and could play a role in balancing the epithelial and mesenchymal attractors.

Supplementary Note 4: Constraints of the Gene Ontology-based functional characterization of our compartmentalized model

The results of the Gene Ontology based functional analysis show (Supplementary Figure 6), that during EMT we could observe an activation of the "epithelial to mesenchymal transition" process as well as the activation of "cell motility" as expected. These observations are in concert with known biological observations and they are important to functionally characterize our model.

On the other hand, this analysis couldn't recapitulate the fact that cell-cell and cell-matrix adhesion weaken during EMT. There was a marked decrease at the middle of the transition but almost immediately these functions were activated again. These contradictory results stemmed from the fact that our network is compartmentalized but the GO database is not. The GO database annotates proteins with biological terms, this neglects the fact that proteins are often functioning compartment-specifically. In our model this results in a false readout, as an example the activation of Ecadherin_CTF still contributes to the "epithelial cell-cell adhesion" activity which is invalid from a biological perspective. Thus to appropriately characterize the functional changes of a compartmentalized model future studies must use a compartmentalized functional readout which is not available yet.

Supplementary Note 5: Correlation of node compartmentalization with protein mass and function

In our compartmentalized EMT model there are divided nodes that represent translocating proteins according to subcellular locations (see Fig. 2 of the main text). An important difference of our model to the original EMT model is that in our case the nodes that represent one protein do not always mutually inhibit each other. This results in the possibility, that one protein has simultaneous activity in different organelles. One could argue that this breaks the law of mass conservation, as simultaneous activation in more than one organelle assumes increased protein mass. In Boolean models the base hypothesis is that there's a threshold above which there's an observable function, but this threshold may differ in different organelles. One underlying reason is that the functionality of a protein is not always proportionate with its concentration. If a protein translocates to an organelle in which there are interaction partners of that given protein with high affinity, then a relatively small amount of translocated proteins can have functionality and the original function in the other organelle may be preserved. Also the volume of organelles varies greatly e.g.: the cytoplasm is relatively large while the nucleus or mitochondria are notably smaller not to mention biomolecular condensates[7,8]. A flow of a small amount of proteins from a large organelle to a smaller is enough to reach locally high concentration levels. Moreover, in biomolecular systems the protein mass is dynamically regulated, proteins may be degraded or synthesized, all these also

result in the alteration of protein mass. Thus the simultaneously active Boolean state of a protein in more organelles does not by any means implies increased overall protein mass.

Supplementary Figures

Supplementary Figure 1: Algorithmic operations and program modules of our workflow

Although we created a supporting website (https://translocaboole.linkgroup.hu) for our project (where a basic analysis with preset options can be run), it is also possible to run the analysis codes accessing them through our GitHub repository (https://github.com/deriteidavid/compartmentalized_EMT_Boolean_model_Mendik_et_al_2021) **a.** Using the Translocatome database (www.translocatome.linkgroup.hu) it's possible to predict the translocation probability of a given protein. **b.** After that it's possible to start a guided manual curation process which focuses on predicted translocating proteins. **c.** After these two initial manual steps all following steps are automated. Based on the curated information the compartmentalized Boolean rules can be generated *via* our website, and the files EMT_E.txt and EMT_M.txt can be downloaded for further analysis (or users can choose to run the analysis according to the default options provided on the website). EMT_E and EMT_M files contain at the top of the files the state of all nodes in the epithelial and in the mesenchymal state respectively and after that all the Boolean rules of the model **d.** The BooleanNet program (found in the BooleanNet folder of the GitHub repository) uses the EMT_E and EMT_M files as input for the dynamic simulations and also a pathway_mapping.xlsx file (which maps the proteins to their respective signalling pathways). The content of the output files is the following: *Final_states.xlsx*: lists the activity of each node in the final state after each perturbation that was introduced to the system. *Trajectories.xlsx*: shows which protein changes its activity in a given timestep during a simulation. *Initial_states.xlsx*: lists the activity of each node at the beginning of each perturbation. Pathway activities files are generated for every introduced perturbation, and the naming of the file contains the perturbed node. The plot visualizes the activity of the signalling pathways (similar to Figure 5). *Attractor_states.png*: this is a visual representation of the Final_states.xlsx file, this file visualizes the arising attractors (similarly as on Supplementary Figure 3 or 4), where each attractor state arising from perturbations introduced to the epithelial state are shown. **e.** The Jupyter

notebook contained in the attractor_repertoire_and_stable_motif_analysis folder calculates the stable motifs and the control sets of a model according to the method of Rozum et al.[4] This code uses only the Boolean rules of a model (from files EMT_E.txt or EMT_M.txt), the definition of the initial states is not required. **f.** Similarly, another Jupyter notebook in the attractor_probabilities folder calculates the stability of different attractors. There's also no need for defined initial states, Boolean rules are enough (from files EMT_E or EMT_M).

Sample input files are available on the GitHub repository along with the codes.

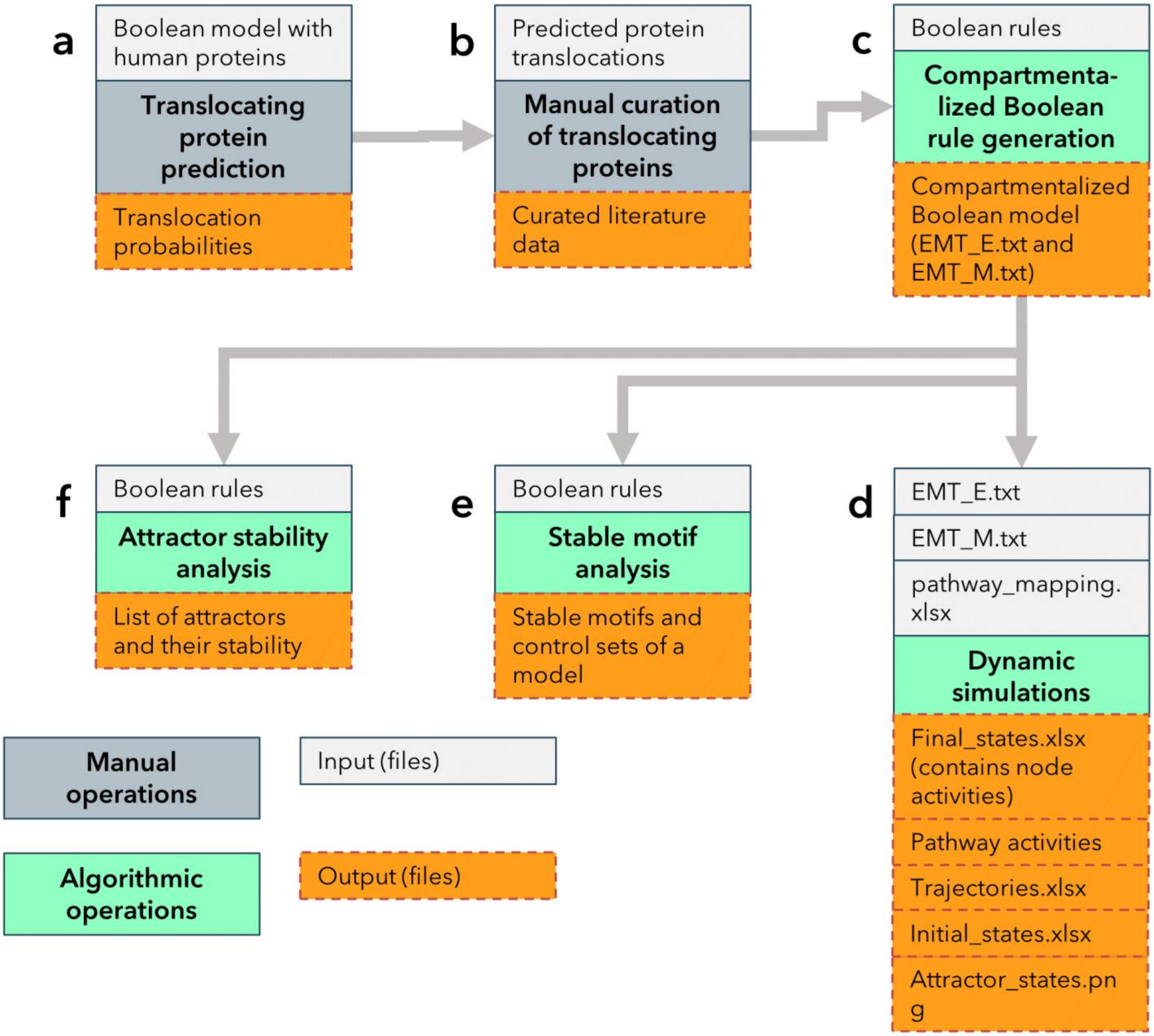

Supplementary Figure 2: Stable motif succession diagram of the new compartmentalized model

Nodes represent stable (top row) and conditionally stable motifs, and in the bottom line the attractors. For reference on the attractors and the node labels see Supplementary Tables 1 and 2, respectively. Directed edges represent possible successive stabilizations of motifs. Orange coloured nodes represent motifs consistent with the mesenchymal attractor (M) and inconsistent with E, blue coloured nodes represent motifs consistent with the epithelial attractor (E) and inconsistent with M. Green nodes represent motifs, which are mesenchymal but are consistent with some epithelial motifs (blue). The attractors coloured with green all contain at least one hybrid motif. The green edges show all possible successive stabilisation paths that lead to the epithelial attractor.

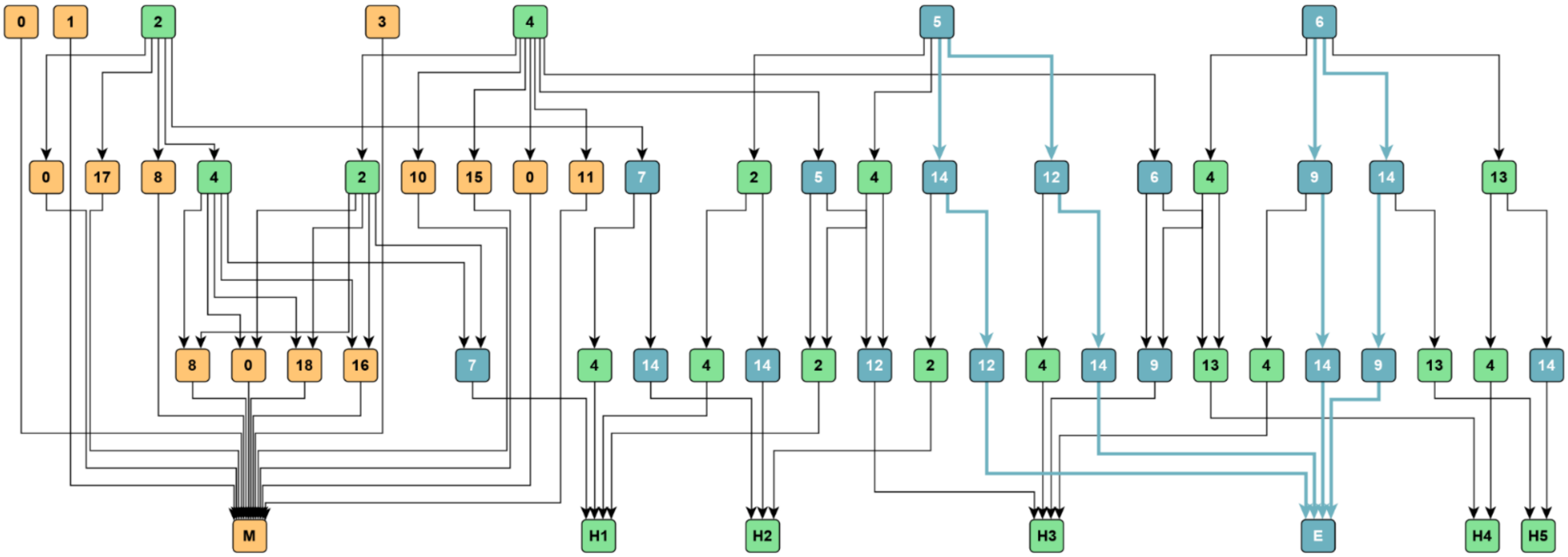

Supplementary Figure 3: Stable states of the original EMT model after single node perturbations

On this figure we show the result of single node KI/KO perturbations. Each column represents the steady state (attractor) of the model where it stabilized after a one node perturbation. The column's name is the perturbed node's name. Each row shows a node in the model and one can assess its state in different perturbations. The nodes' colour indicates if the node's state corresponds to the epithelial or the mesenchymal attractor, blue colours are epithelial, whereas orange colours are mesenchymal node states. The text indicates the exact Boolean state of a node, either TRUE or FALSE.

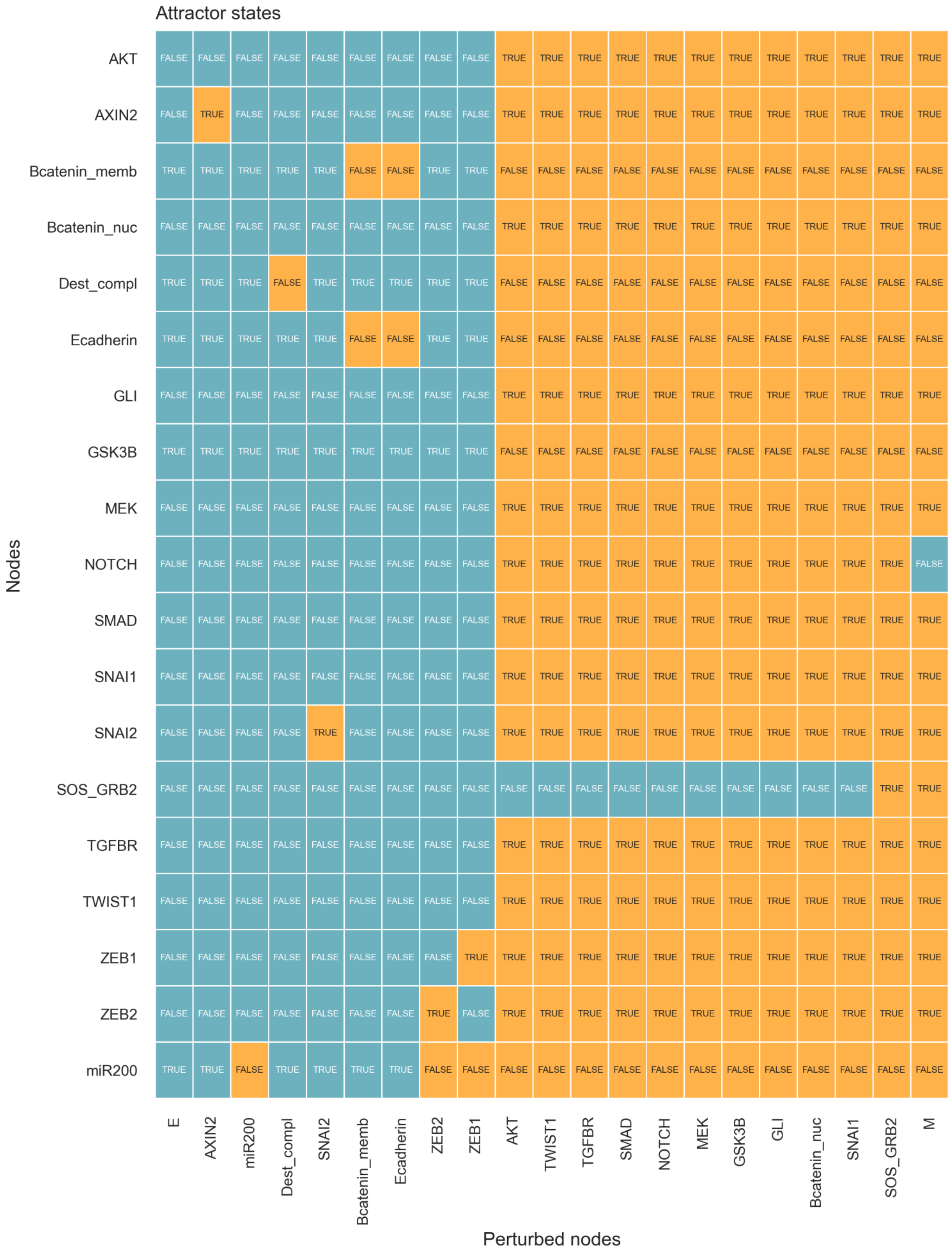
Attractor states
Nodes
AKT
AXIN2
Bcatenin_memb
Bcatenin_nuc
Dest_compl
Ecadherin
GLI
GSK3B
MEK
NOTCH
SMAD
SNAI1
SNAI2
SOS_GRB2
TGFBR
TWIST1
ZEB1
ZEB2
miR200
E
AXIN2
miR200
Dest_compl
SNAI2
Bcatenin_memb
Ecadherin
ZEB2
ZEB1
AKT
TWIST1
TGFBR
SMAD
NOTCH
MEK
GSK3B
GLI
Bcatenin_nuc
SNAI1
SOS_GRB2
M
Perturbed nodes

Supplementary Figure 4: Stable states of the compartmentalized EMT model after single node perturbations

On this figure we show the result of single node KI/KO perturbations. Each column represents the steady state (attractor) of the model where it stabilized after a one node perturbation. The column's name is the perturbed node's name. Each row shows a node in the model and one can assess its state in different perturbations. The nodes' colour indicates if the node's state corresponds to the epithelial or the mesenchymal attractor, blue colours are epithelial, whereas orange colours are mesenchymal node states. The text indicates the exact Boolean state of a node, either TRUE or FALSE.

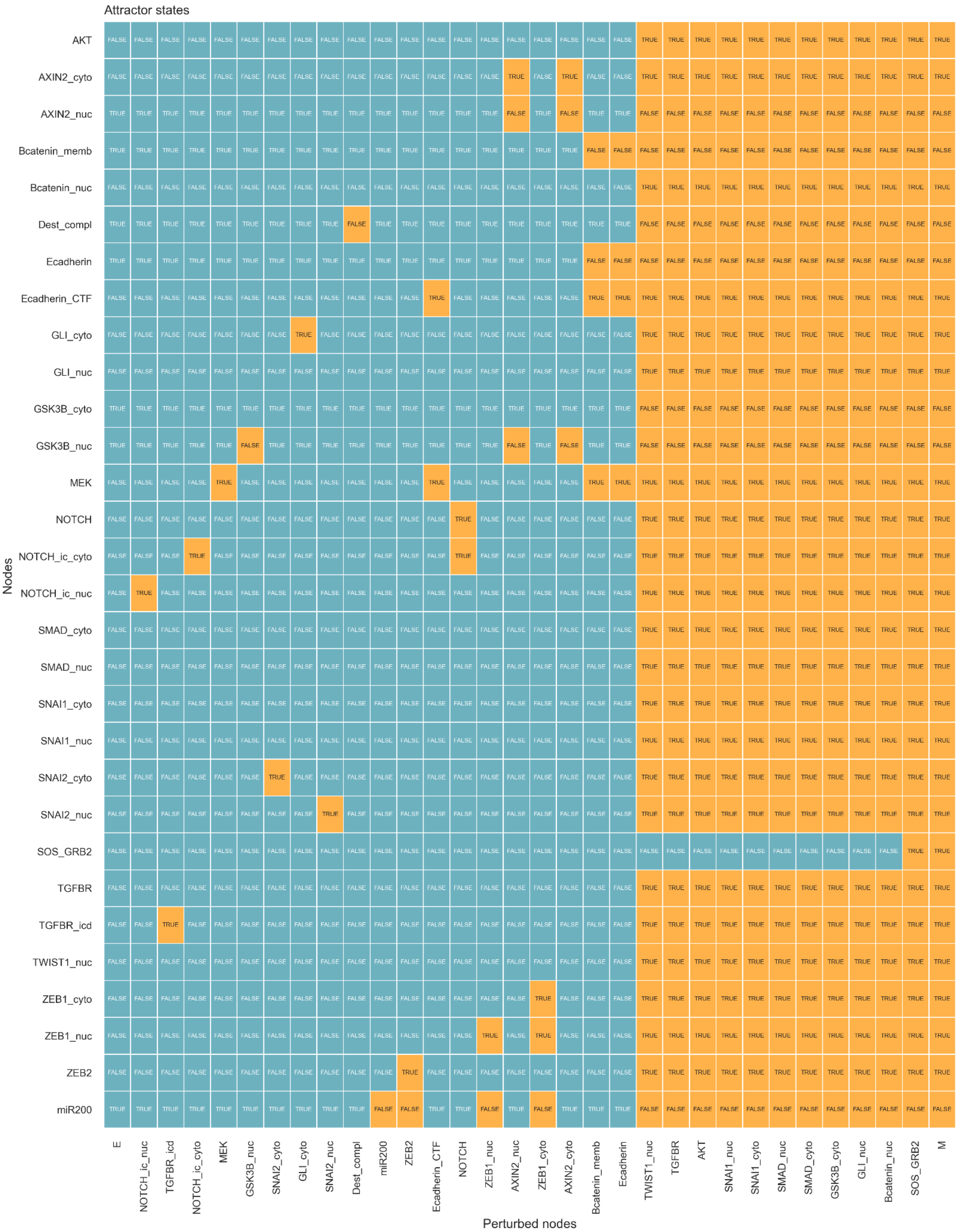
Attractor states
Nodes
AKT
AXIN2_cyto
AXIN2_nuc
Bcatenin_memb
Bcatenin_nuc
Dest_compl
Ecadherin
Ecadherin_CTF
GLI_cyto
GLI_nuc
GSK3B_cyto
GSK3B_nuc
MEK
NOTCH
NOTCH_ic_cyto
NOTCH_ic_nuc
SMAD_cyto
SMAD_nuc
SNAI1_cyto
SNAI1_nuc
SNAI2_cyto
SNAI2_nuc
SOS_GRB2
TGFBR
TGFBR_icd
TWIST1_nuc
ZEB1_cyto
ZEB1_nuc
ZEB2
miR200
E
NOTCH_ic_nuc
TGFBR_icd
NOTCH_ic_cyto
MEK
GSK3B_nuc
SNAI2_cyto
GLI_cyto
SNAI2_nuc
Dest_compl
miR200
ZEB2
Ecadherin_CTF
NOTCH
ZEB1_nuc
AXIN2_nuc
ZEB1_cyto
AXIN2_cyto
Bcatenin_memb
Ecadherin
TWIST1_nuc
TGFBR
AKT
SNAI1_nuc
SNAI1_cyto
SMAD_nuc
SMAD_cyto
GSK3B_cyto
GLI_nuc
Bcatenin_nuc
SOS_GRB2
M
Perturbed nodes

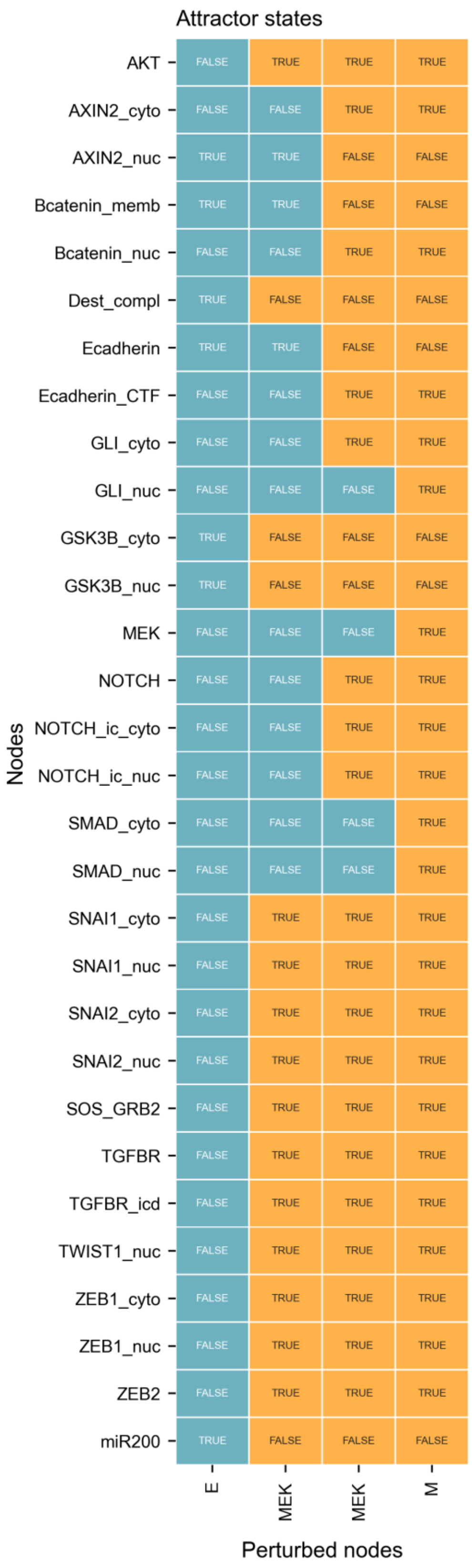

Supplementary Figure 5: Uncovering the effect of MEK KO with TGFBR ON simulations

On this figure we show the result of single node KI/KO perturbations. Each column represents the steady state (attractor) of the model where it stabilized after a one node perturbation. The column's name is the perturbed node's name. Each row shows a node in the model and one can assess its state in different perturbations. The nodes' colour indicates if the node's state corresponds to the epithelial or the mesenchymal attractor, blue colours are epithelial whereas orange colours are mesenchymal node states. The text indicates the exact Boolean state of a node, either TRUE or FALSE.

In this simulation MEK KO results in either one of the two middle attractors. The left one is a more epithelial like attractor with E-cadherin expression. During the simulations this attractor was reached in 88% of the cases.

The right attractor from the middle two is more mesenchymal with no E-cadherin expression, but the simulations only resulted in this attractor in 12% of the cases.

Supplementary Figure 6: Activity of 5 GO biological processes during EMT

Here we show the activity of 5 GO biological processes during EMT. There are 11 single node perturbations that led to EMT, here we show the average activation of these GO terms during these 11 perturbations. The figure shows the activation the “epithelial to mesenchymal transition” and “cell motility” processes during EMT. In case of the other processes the results are contradictory as the marked decrease in activity is followed by an instant activation.

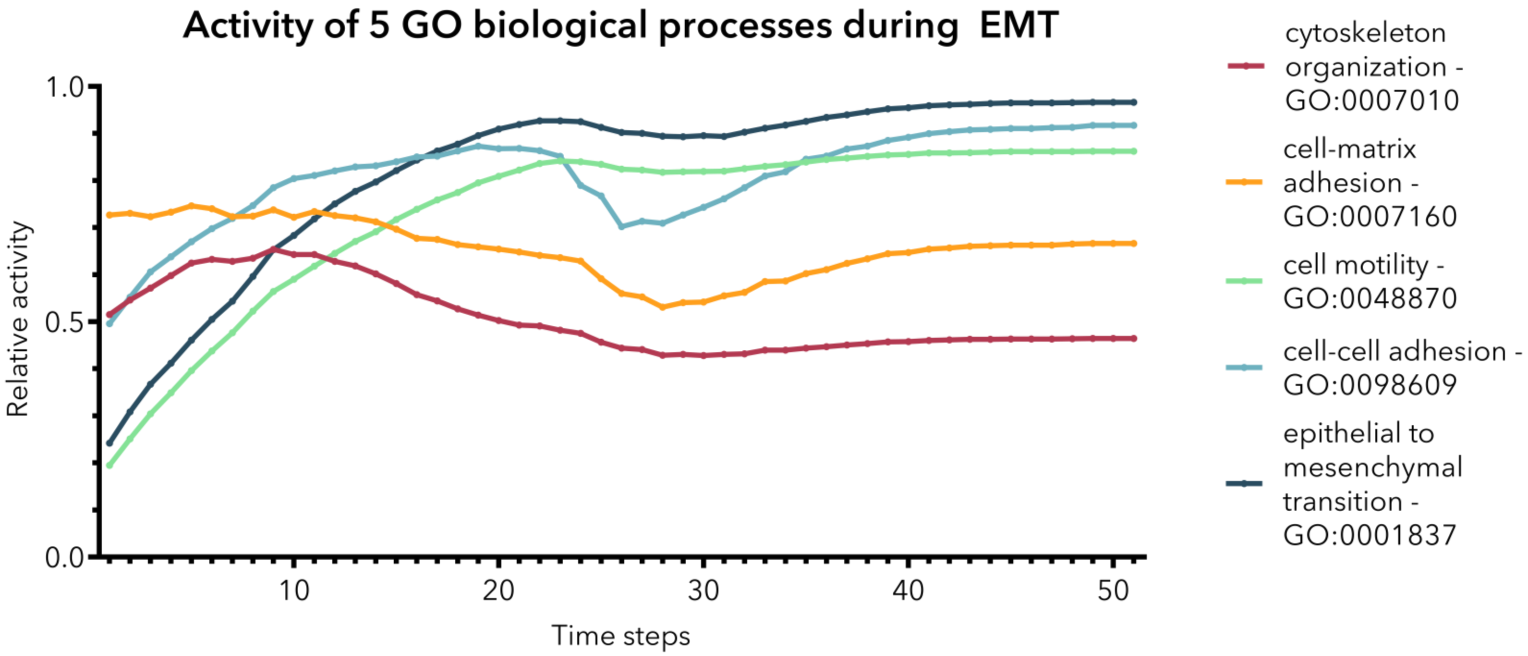

Supplementary Figure 7: All possible node compartmentalization scenarios

A compartmentalized node (separated to two different nodes) could be represented in several ways, but there are 3 main underlying principles. In the first case there is a one-way communication between the compartmentalized nodes, either inhibitory or activatory, these are reflected in scenarios a, b, c, d. In these cases there's no direct feedback between the compartmentalized nodes. Scenario e happens when the different pools of a protein are regulated separately and there's no direct interaction between those protein pools. In scenarios f, g, h and i there's direct feedback between the compartmentalized nodes and these could be either inhibitory or activatory or a combination of both. As highlighted with blue rectangles, nodes NOTCH, SMAD, SNAI1, TGFBR, ZEB1 were compartmentalized according to a, Ecadherin according to c, GSK3B according to e, AXIN2 and Bcatenin according to f and lastly GLI and SNAI2 according to g.

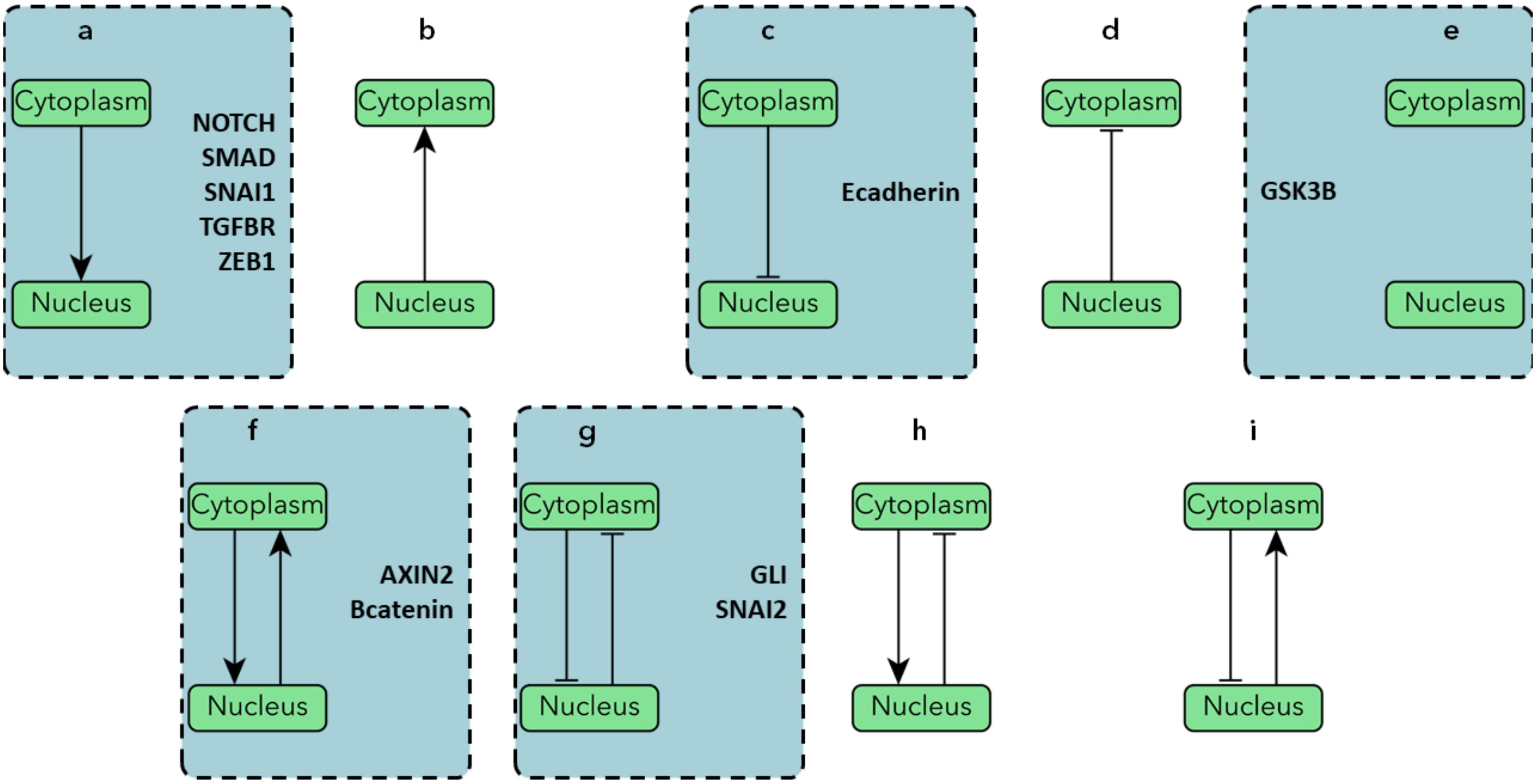

Supplementary Tables

Supplementary Table 1: Attractors of the compartmentalized model

This table shows all the possible attractors of our compartmentalized model. Each column shows an attractor where one line shows the Boolean value of a certain node. Attractor 0 is the mesenchymal attractor and attractor 5 is the epithelial phenotype. Other attractors (marked with H) are hybrid attractors. We also incorporated the attractor stability, the Hamming distance and the Normalized overlap in this table.

	Mesenchymal attractor - M	H1	H2	H3	H4	H5	Epithelial attractor - E
	**0**	**1**	**4**	**2**	**3**	**6**	**5**
**AKT**	1	0	0	0	0	0	0
**AXIN2_cyto**	1	1	0	1	1	0	0
**AXIN2_nuc**	0	0	1	0	0	1	1
**Bcatenin_memb**	0	0	0	1	1	1	1
**Bcatenin_nuc**	1	0	0	0	0	0	0
**Dest_compl**	0	1	1	1	0	0	1
**Ecadherin**	0	0	0	1	1	1	1
**Ecadherin_CTF**	1	1	1	0	0	0	0
**GLI_cyto**	1	0	0	0	0	0	0
**GLI_nuc**	1	0	0	0	0	0	0
**GSK3B_cyto**	0	1	1	1	1	1	1
**GSK3B_nuc**	0	0	1	0	0	1	1
**MEK**	1	1	1	0	0	0	0
**NOTCH**	1	0	0	0	0	0	0
**NOTCH_ic_cyto**	1	0	0	0	0	0	0
**NOTCH_ic_nuc**	1	0	0	0	0	0	0
**SMAD_cyto**	1	0	0	0	0	0	0
**SMAD_nuc**	1	0	0	0	0	0	0
**SNAI1_cyto**	1	0	0	0	0	0	0
**SNAI1_nuc**	1	0	0	0	0	0	0
**SNAI2_cyto**	1	0	0	0	0	0	0
**SNAI2_nuc**	1	0	0	0	0	0	0
**SOS_GRB2**	0	0	0	0	0	0	0
**TGFBR**	1	0	0	0	0	0	0
**TGFBR_icd**	1	0	0	0	0	0	0
**TWIST1_nuc**	1	0	0	0	0	0	0
**ZEB1_cyto**	1	0	0	0	0	0	0
**ZEB1_nuc**	1	0	0	0	0	0	0
**ZEB2**	1	0	0	0	0	0	0
**miR200**	0	1	1	1	1	1	1
**Attractor stability**	0,945675	0,000539	0,000900	0,000131	0,000289	0,001645	0,000437
**Hamming distance from E attractor**	29	7	4	3	4	2	0
**Normalized overlap with E attractor**	0,03	0,77	0,87	0,90	0,87	0,93	1,00

Supplementary Table 2: List of stable and conditionally stable motifs in the compartmentalized model

These are the stable motifs of the compartmentalized model (including conditionally stable motifs), which were identified during the stable motif analysis as detailed in Supplementary Note 1.

index	(Conditionally) Stable Motif
0	AKT=1, GSK3B_cyto=0
1	GSK3B_nuc=0, AKT=1, Bcatenin_memb=0, NOTCH_ic_cyto=1, NOTCH=1, Bcatenin_nuc=1
2	Bcatenin_memb=0, Ecadherin=0
3	GSK3B_nuc=0, ZEB1_nuc=1, AKT=1, ZEB1_cyto=1, SMAD_cyto=1, SNAI1_cyto=1, SMAD_nuc=1, SNAI1_nuc=1, TGFBR=1, MEK=1, miR200=0
4	AXIN2_cyto=1
5	GSK3B_cyto=1, AKT=0, SMAD_cyto=0, Dest_compl=1, NOTCH_ic_cyto=0, SMAD_nuc=0, SNAI1_nuc=0, NOTCH=0, SNAI1_cyto=0, GLI_nuc=0, TGFBR=0, Bcatenin_nuc=0, TWIST1_nuc=0, SOS_GRB2=0
6	GSK3B_cyto=1, AKT=0, SMAD_cyto=0, SMAD_nuc=0, SNAI1_nuc=0, Bcatenin_memb=1, SNAI1_cyto=0, GLI_nuc=0, Ecadherin=1, Bcatenin_nuc=0, TGFBR=0, TWIST1_nuc=0, SOS_GRB2=0
7	GSK3B_cyto=1, AKT=0, SMAD_cyto=0, Dest_compl=1, NOTCH_ic_cyto=0, SMAD_nuc=0, SNAI1_nuc=0, NOTCH=0, SNAI1_cyto=0, GLI_nuc=0, TGFBR=0, Bcatenin_nuc=0, TWIST1_nuc=0
8	Bcatenin_nuc=1, NOTCH_ic_cyto=1, NOTCH=1
9	Dest_compl=1
10	GLI_cyto=1, ZEB1_nuc=1, ZEB1_cyto=1, SMAD_cyto=1, SNAI1_cyto=1, SMAD_nuc=1, SNAI1_nuc=1, GLI_nuc=1, TGFBR=1, MEK=1, GSK3B_cyto=0, miR200=0
11	Bcatenin_memb=0, Bcatenin_nuc=1, NOTCH_ic_cyto=1, NOTCH=1
12	Ecadherin=1, Bcatenin_memb=1
13	Dest_compl=0
14	AXIN2_nuc=1, AXIN2_cyto=0
15	ZEB1_nuc=1, AKT=1, ZEB1_cyto=1, SMAD_cyto=1, SNAI1_cyto=1, SMAD_nuc=1, SNAI1_nuc=1, TGFBR=1, MEK=1, miR200=0
16	ZEB1_nuc=1, AKT=1, ZEB1_cyto=1, SMAD_cyto=1, SNAI1_cyto=1, SMAD_nuc=1, SNAI1_nuc=1, TGFBR=1, miR200=0
17	GSK3B_nuc=0, ZEB1_nuc=1, AKT=1, ZEB1_cyto=1, SMAD_cyto=1, SNAI1_cyto=1, SMAD_nuc=1, SNAI1_nuc=1, TGFBR=1, miR200=0
18	GLI_cyto=1, ZEB1_nuc=1, ZEB1_cyto=1, SMAD_cyto=1, SNAI1_cyto=1, SMAD_nuc=1, SNAI1_nuc=1, GLI_nuc=1, TGFBR=1, GSK3B_cyto=0, miR200=0

Supplementary Table 3: Attractor stability in case of the epithelial and mesenchymal attractors in the compartmentalized and in the original EMT model

This table compares the attractor stability measures of our compartmentalized model and the original model. These values were calculated as detailed in the attractor stability anyalsis subsection of the methods (main text).

	Attractor stability	
	**Epithelial attractor**	**Mesenchymal attractor**
**Compartmentalized EMT model**	0,94567534	0,000437285
**Original EMT model**	0,974082334	0,000109385

Supplementary Table 4: Experimental outcomes reproduced by the compartmentalized and the original EMT model

The publication of Steinway et al.[3] used the experimental results detailed in this table to prove the righteousness of their model. To ensure our compartmentalized model performs similarly we observed the same experimental results and concluded that it reproduces all experimental results as expected.

Experimental result	Reference (PMID)	Recaptured by the original EMT model	Recaptured by the compartmentalized model
TGFβ signalling leads to SMAD complex formation, MAP kinase signalling, and AKT signalling.	20495575	yes	Yes
Wnt signalling leads to nuclear localization of β-catenin, AXIN2 induction, and suppression of the destruction complex	11809808	yes	Yes
SHH signalling leads to induction of GLI transcription factors	21789137	yes	Yes
miR200 inhibits TGFβ–driven EMT	18411277	yes	Yes
E-cadherin suppressing transcription factors SNAI1, SNAI2, ZEB1, ZEB2 and TWIST1 induce EMT when acting together	22945800, 20713713	yes	Yes
Constitutive SNAI1 or TWIST1 activation drives EMT	22945800, 20713713, 21199805, 21317430	yes	Yes

Supplementary Table 5: Nodes annotated to signalling pathways

We've annotated each node of compartmentalized network to a signalling pathway in order to observe signalling activities during EMT. These signalling pathways are inactive at the beginning of EMT and they get activated during the transition. Their inactive state is marked in the last column.

Node	Annotated signalling pathway	Node state in the epithelial attractor
AKT	TGFβ-BMP SMAD independent	FALSE
AXIN2_cyto	WNT	FALSE
AXIN2_nuc		TRUE
Bcatenin_memb	WNT	TRUE
Bcatenin_nuc		FALSE
Dest_compl	WNT	TRUE
Ecadherin	WNT	TRUE
Ecadherin_CTF		FALSE
GLI_cyto	Hedgehog	FALSE
GLI_nuc		FALSE
GSK3B_nuc	WNT	TRUE
GSK3B_cyto		TRUE
MEK	Receptor tyrosine kinase	FALSE
NOTCH	Notch	FALSE
NOTCH_ic_cyto		FALSE
NOTCH_ic_nuc		FALSE
SMAD_cyto	TGFβ-BMP SMAD dependent	FALSE
SMAD_nuc		FALSE
SNAI1_cyto	TGFβ-BMP SMAD dependent	FALSE
SNAI1_nuc		FALSE
SNAI2_cyto	TGFβ-BMP SMAD dependent	FALSE
SNAI2_nuc		FALSE
SOS_GRB2	Receptor tyrosine kinase	FALSE
miR200	TGFβ-BMP SMAD dependent	TRUE
TGFBR	TGFβ-BMP SMAD dependent and independent	FALSE
TGFBR_icd		FALSE
TWIST1_nuc	Hypoxia	FALSE
ZEB1_cyto	TGFβ-BMP SMAD dependent	FALSE
ZEB1_nuc		FALSE
ZEB2	TGFβ-BMP SMAD dependent	FALSE

Supplementary References

1 Zañudo, J. G. T. & Albert, R. An effective network reduction approach to find the dynamical repertoire of discrete dynamic networks. *Chaos: An Interdisciplinary Journal of Nonlinear Science* **23**, 025111, (2013).

2 Deritei, D., Rozum, J., Ravasz Regan, E. & Albert, R. A feedback loop of conditionally stable circuits drives the cell cycle from checkpoint to checkpoint. *Scientific Reports* **9**, 16430, (2019).

3 Steinway, S. N. *et al.* Network modeling of TGFbeta signaling in hepatocellular carcinoma epithelial-to-mesenchymal transition reveals joint sonic hedgehog and Wnt pathway activation. *Cancer Res* **74**, 5963-5977, (2014).

4 Rozum, J. C., Gomez Tejeda Zanudo, J., Gan, X., Deritei, D. & Albert, R. Parity and time reversal elucidate both decision-making in empirical models and attractor scaling in critical Boolean networks. *Sci Adv* **7**, (2021).

5 Zañudo, J. G. T. & Albert, R. Cell Fate Reprogramming by Control of Intracellular Network Dynamics. *PLOS Computational Biology* **11**, e1004193, (2015).

6 Font-Clos, F., Zapperi, S. & La Porta, C. A. M. Topography of epithelial-mesenchymal plasticity. *Proc Natl Acad Sci U S A* **115**, 5902-5907, (2018).

7 Banani, S. F., Lee, H. O., Hyman, A. A. & Rosen, M. K. Biomolecular condensates: organizers of cellular biochemistry. *Nature Reviews Molecular Cell Biology* **18**, 285-298, (2017).

8 Halestrap, A. P. in *Advances in Comparative and Environmental Physiology: Interaction of Cell Volume and Cell Function Volume 14* (eds Florian Lang & Dieter Häussinger) 279-307 (Springer Berlin Heidelberg, 1993).
